# Supplementary material for: Towards a molecular picture of the archaeal cell surface
Source: Nat Commun. 2024 Nov 29;15:10401. doi: 10.1038/s41467-024-53986-9 (PMC11607397; doi:10.1038/s41467-024-53986-9)
Supplement: Supplementary file 1 — Supplementary Information [file 41467_2024_53986_MOESM1_ESM.pdf]

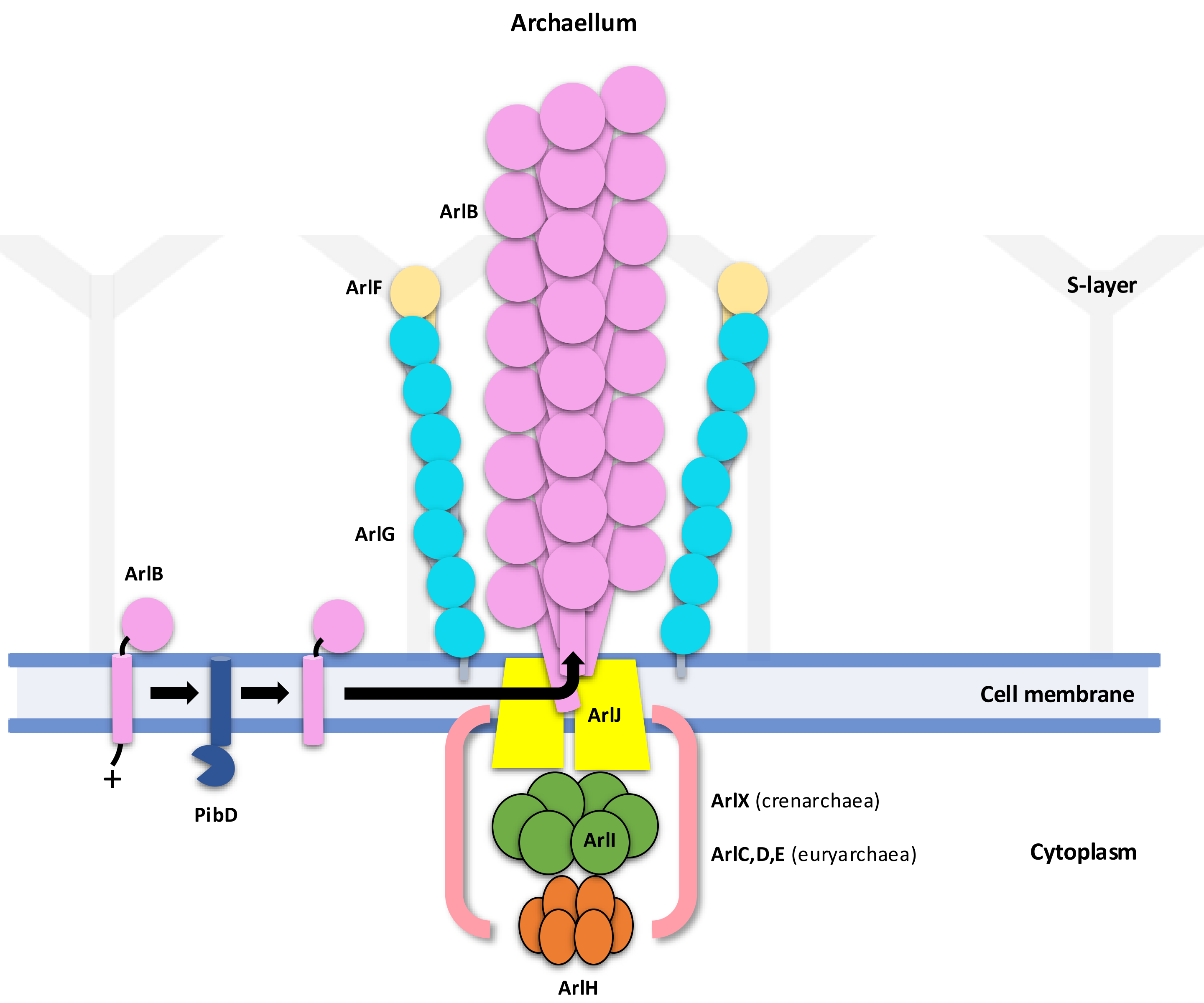
Archaellum
ArlB
ArlF
S-layer
ArlG
ArlB
Cell membrane
ArlJ
PibD
ArlX (crenarchaea)
ArlI
ArlC,D,E (euryarchaea)
Cytoplasm
ArlH

Supplementary Figure 1 – Model of the archaellum machinery

The archaellum machinery is integrated into the cell membrane and S-layer. The archaellum filament is composed of archaellins (pink; ArlB in case of *Sulfolobus acidocaldarius*). The archaellins are initially expressed as membrane-integral proteins via the Sec translocon (not shown). These proteins are then processed by a type-III signal peptidase (PibD), which cleaves the positively-charged N-terminal leader peptide from the archaellin. This primes the archaellin for the integration into the nascent filament, which is assembled by the membrane-integral platform protein ArlJ (yellow). The energy for the assembly of the filament is provided by ATP hydrolysis catalysed by the ATPase ArlI (green). ArlI interacts with ArH, which is thought to act as a regulator that (once the filament is assembled) switches the machinery from assembly to rotation mode. Surrounding ArlJ,I and H is a ring of ArlX in crenarchaea and ArlC,D,E in euryarchaea, which is thought to have stator function. In euryarchaea, ArlC,D,E is also thought to transmit signals from chemoreceptors to the archaellum machinery. In the periplasm, filaments of ArlG, capped by ArlF, integrate the archaellum machinery into the S-layer and likely act as stators[1].

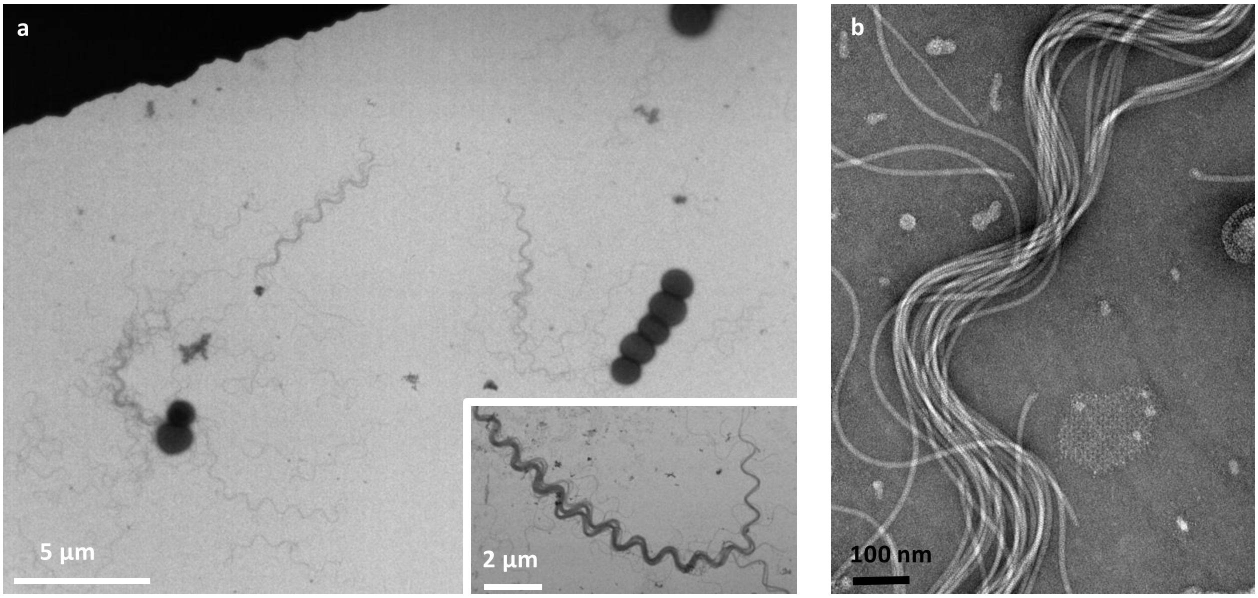
a
5 µm
2 µm
b
100 nm

Supplementary Figure 2 – Example negative stain micrographs of the hyper-archaellated strain MW2106.

a, overview showing various negatively stained cells on the TEM grid. Insert and **b**, magnified views of archaellar bundles associated with the cells.

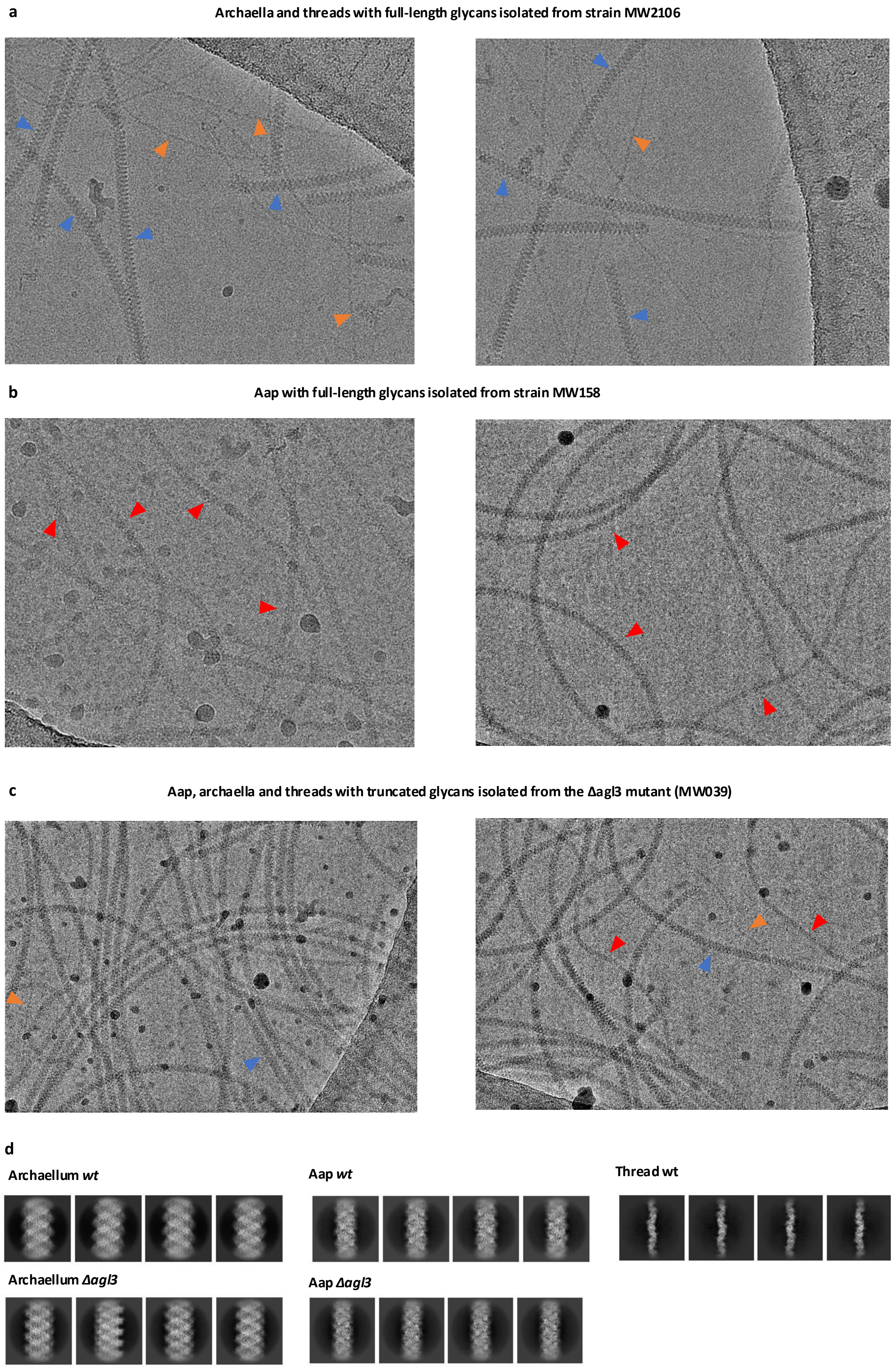
a
Archaella and threads with full-length glycans isolated from strain MW2106
b
Aap with full-length glycans isolated from strain MW158
c
Aap, archaella and threads with truncated glycans isolated from the Δagl3 mutant (MW039)
d
Archaellum *wt*
Aap *wt*
Thread wt
Archaellum *Δagl3*
Aap *Δagl3*

Supplementary Figure 3 – Example cryoEM micrographs and representative 2D classes of isolated filaments from *S. acidocaldarius*.

a-c micrographs of archaella and threads isolated from strain MW2106 (**a**); Aap isolated from strain MW158 (**b**); Aap, archaella and threads isolated from the *Δagl3* mutant (strain MW039) (**c**). Blue arrowheads, archaella; red arrowheads, Aap; orange arrowheads, threads; **b**, representative 2D classes of wt/*Δagl3* archaella, wt/*Δagl3* Aap, and wt threads;

a

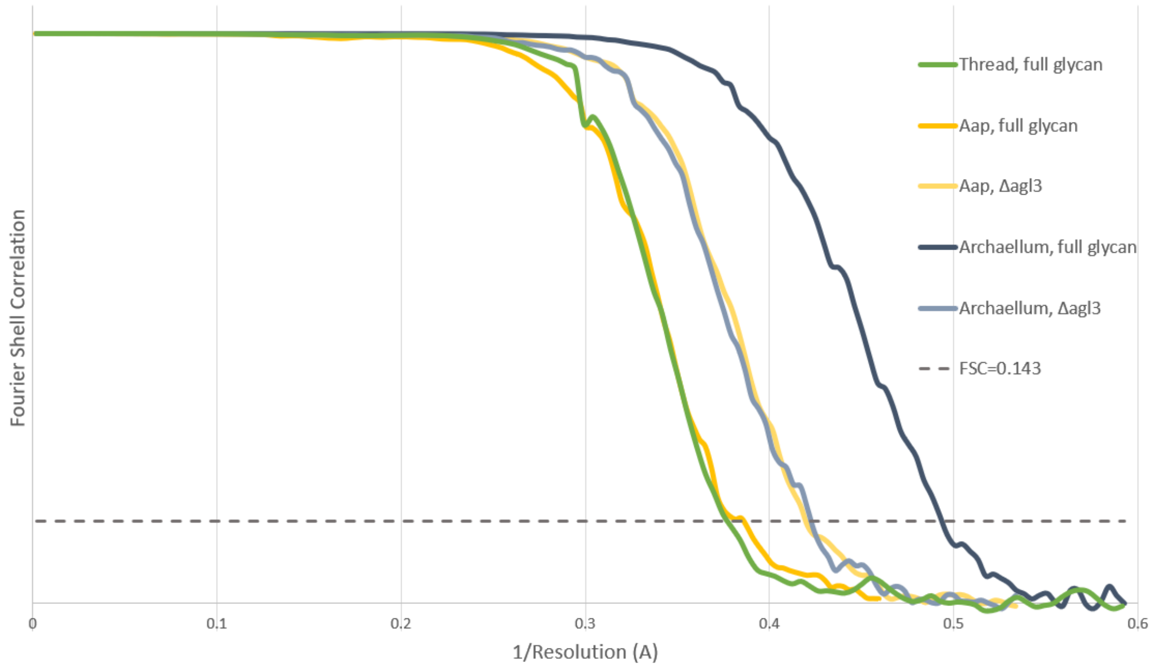
Fourier Shell Correlation
1/Resolution (A)
0
0.1
0.2
0.3
0.4
0.5
0.6
Thread, full glycan
Aap, full glycan
Aap, Δagl3
Archaellum, full glycan
Archaellum, Δagl3
FSC=0.143

b

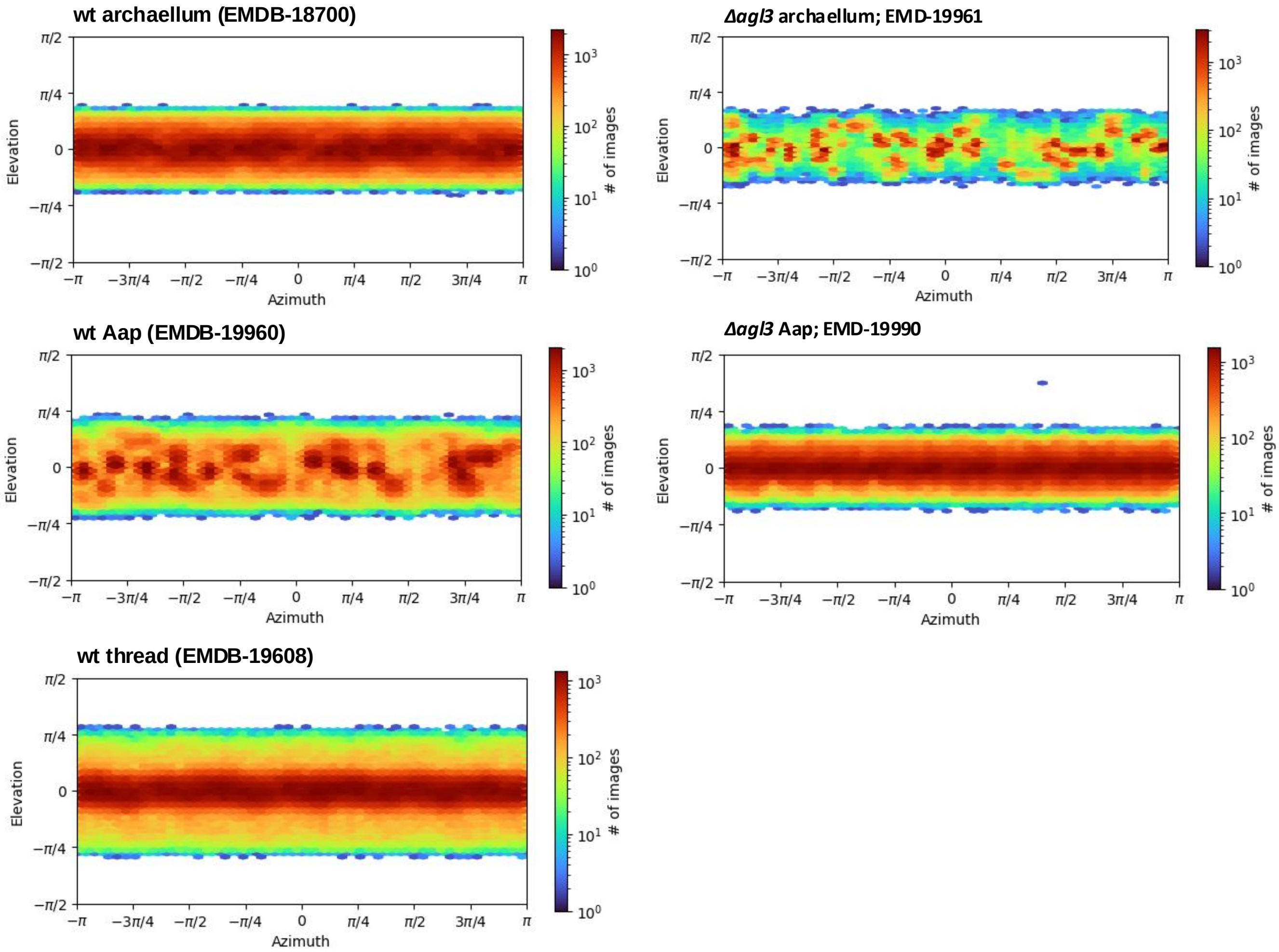
wt archaellum (EMDB-18700)
Δagl3 archaellum; EMD-19961
wt Aap (EMDB-19960)
Δagl3 Aap; EMD-19990
wt thread (EMDB-19608)
Elevation
Azimuth
# of images
π/2
π/4
0
−π/4
−π/2
−π
−3π/4
−π/2
−π/4
π/4
π/2
3π/4
π
10^0
10^1
10^2
10^3

Supplementary Figure 4 – 2D classes, angular sampling and global resolution estimation via Gold Standard Fourier Shell Correlation (FSC).

a, FSC curves for each of the reported cryoEM maps. Filament types are grouped by colour. Thread, green; AAP wt/*Δagl3* mutant, shades of yellow; archaellum wt/*Δagl3* mutant, shades of blue.

b, angular distribution of particles used in the final reconstructions.

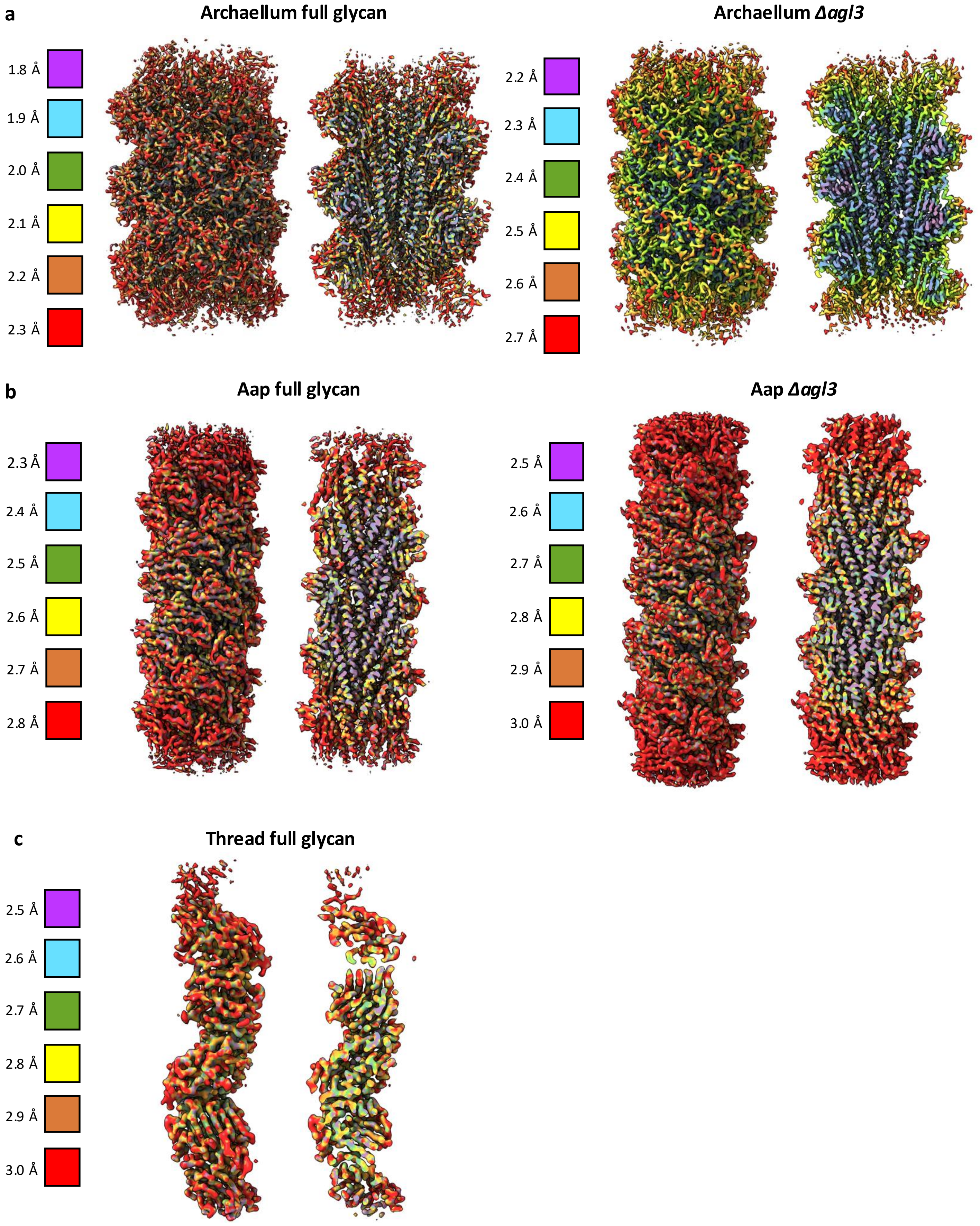
a
Archaellum full glycan
1.8 Å
1.9 Å
2.0 Å
2.1 Å
2.2 Å
2.3 Å
Archaellum Δagl3
2.2 Å
2.3 Å
2.4 Å
2.5 Å
2.6 Å
2.7 Å
b
Aap full glycan
2.3 Å
2.4 Å
2.5 Å
2.6 Å
2.7 Å
2.8 Å
Aap Δagl3
2.5 Å
2.6 Å
2.7 Å
2.8 Å
2.9 Å
3.0 Å
c
Thread full glycan
2.5 Å
2.6 Å
2.7 Å
2.8 Å
2.9 Å
3.0 Å

Supplementary Figure 5 – Local Resolution estimation

a, maps in side view and cross section of the wt archaellum with the full glycan (left) and the archaellum from the Δagl3 mutant (right). **b,** maps in side view and cross section of the wt Aap with the full glycan (left) and the Aap from the Δagl3 mutant (right). **c**, map in side view and cross section of the wt thread with the full glycan

a

Signal peptide

```
MIYMKRRKTRGLAGLDTAIILIAFIITAAVLAYVAVNMGLFVTQKAKTTINKGEETASTA
LSLSGNVLYAVNYPTNTKSYWMYFTVSPSSGVSSVDLSPSTTAISFTAASRGVSLSNIYQ
FSLLSVLPSQVNNKVQVKLGTSIINLTLAFSSNSAGQTYVYYSDPNYALLALNYTLGQEV
KGGQLTSSPLYIISNTSIVASKPWLKNDNVFTFNISVNGTEVEYYAYVNKTFAFTYPVSG
FPLAGSDIAPAGSVIGVMILFGPGEATNVFQYETVTIQITPNIGSPLTISQYIYQPDGKV
TVIG
```

b

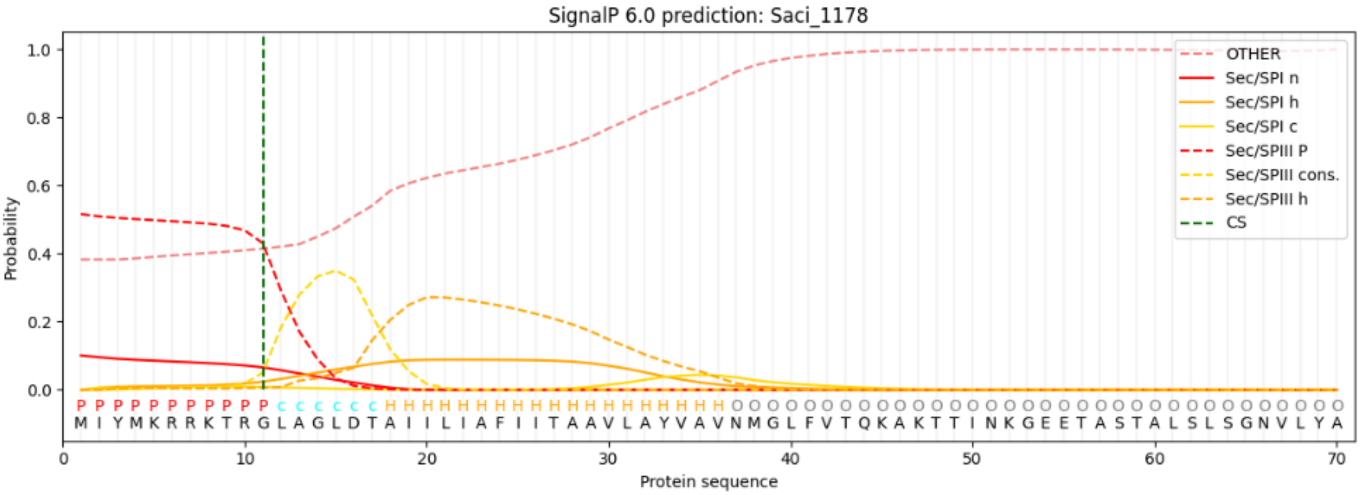

Supplementary Figure 6 – ArlB sequence and Sec-III peptidase

a, amino acid sequence of ArlB. The signal peptide is indicated. The sequence of the mature protein is coloured by domain. The α-helix is red, the main head domain is grey, and the glycosylation sub-domain is cyan. **b,** the SignalP 6.0 server [2] predicts a signal-III peptidase cleavage site.

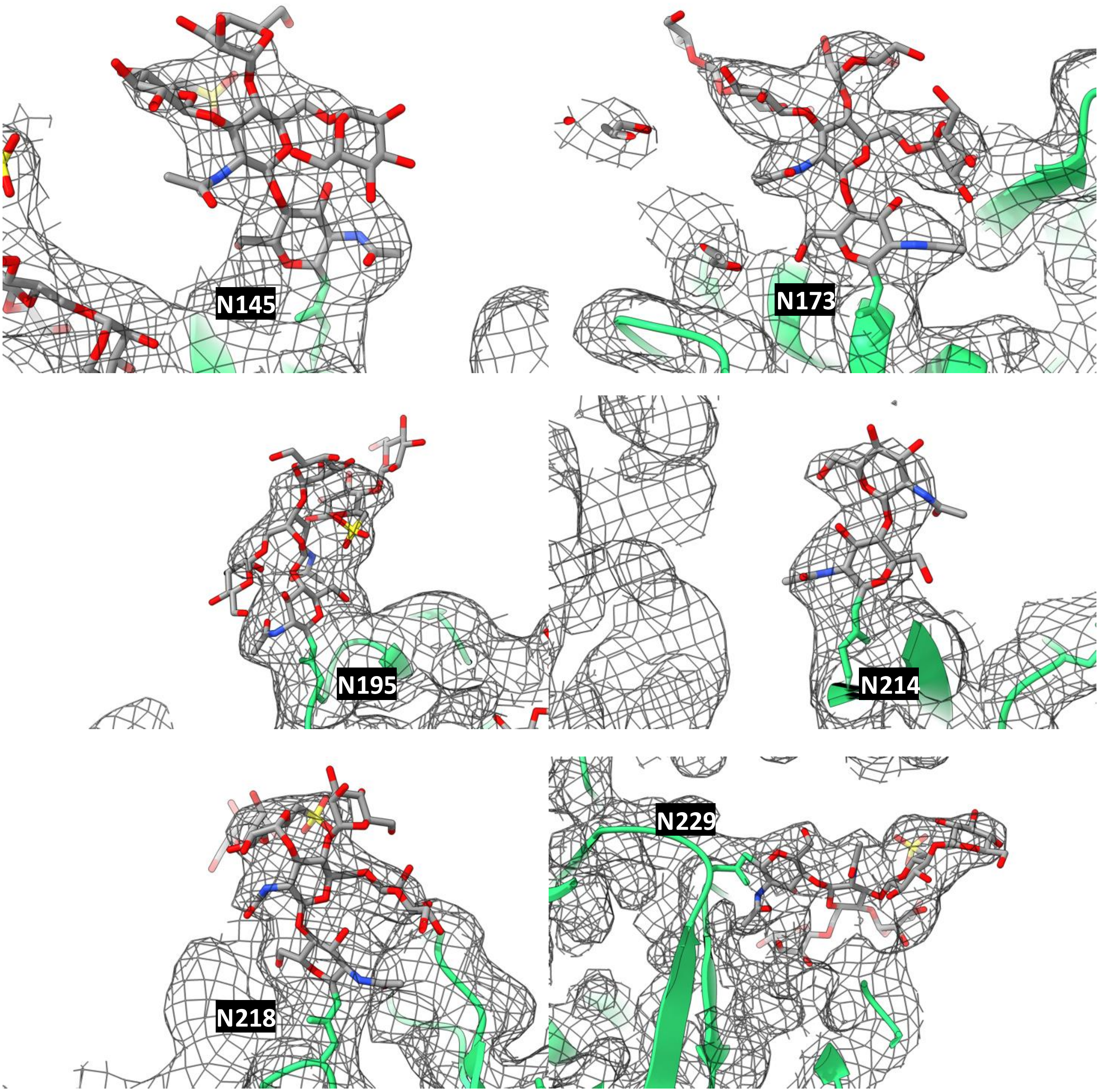
N145
N173
N195
N214
N218
N229

Supplementary Figure 7 – Glycosylation sites within the archaellum

Close-ups of the six glycosylation sites (N145, N173, N195, N214, N218 and N229) within the *S. acidocadarius* archaellum. Protein is shown as green ribbons and glycans as sticks, coloured by element. The cryoEM map is shown as grey mesh.

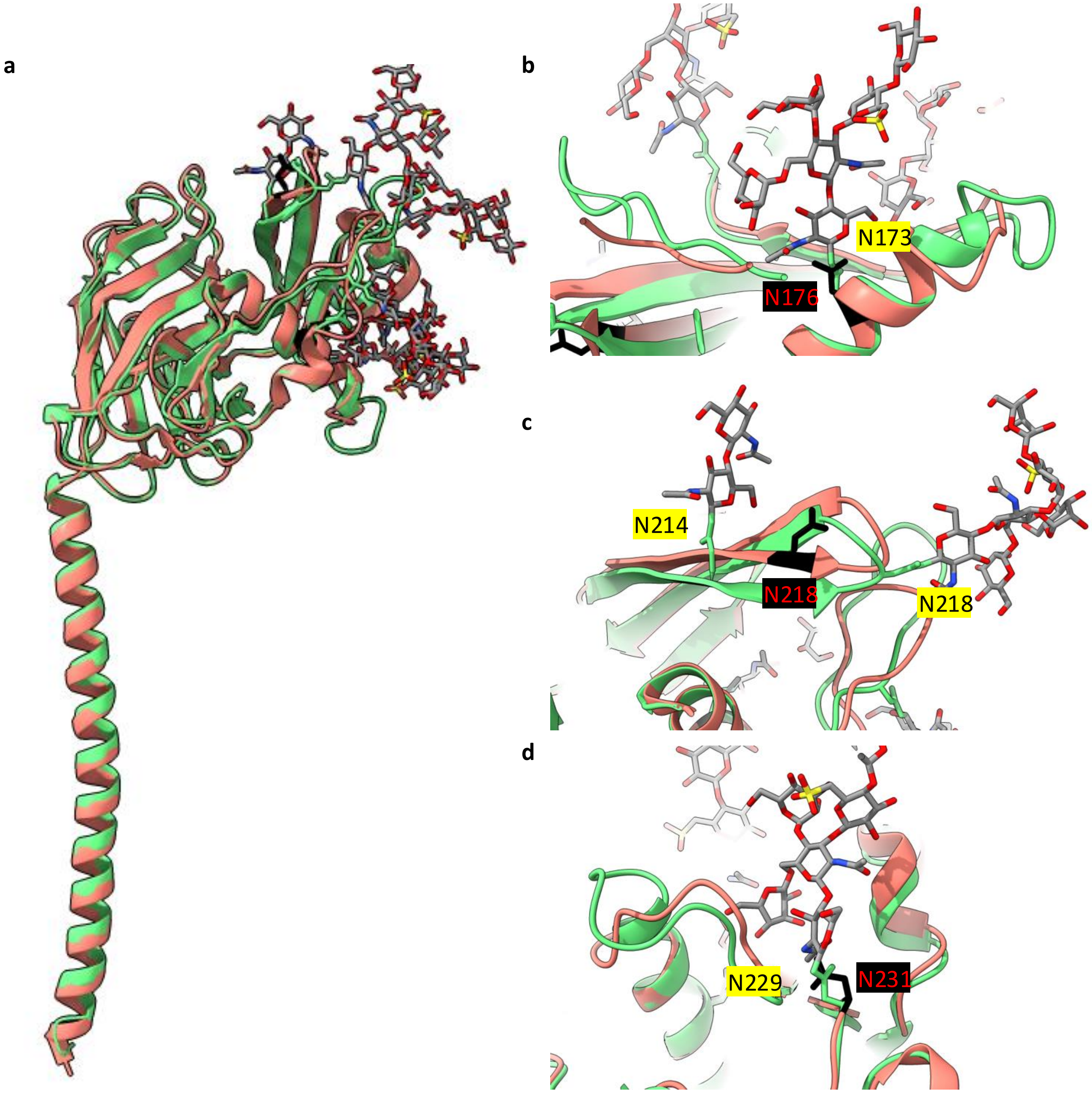
a
b
N173
N176
c
N214
N218
N218
d
N229
N231

Supplementary Figure 8: *Conservation between S. acidocaldarius* and *S. islandicus* ArlB

a, superimposition of the archaellins from *S. acidocaldarius* (green) and *S. islandicus* [3] (PDB-8CWM; salmon) showing almost identical protein structures. However, the glycosylation sites are divergent. All three glycosylation sites in *S. islandicus* (red text on black background) line up with the corresponding sites in *S. acidocalrarius* (black text on yellow background). However, the *S. acidocaldarius* archaellum has three additional glycosylation sites (N145, N195 and 214) that are not found in *S. islandicus.*

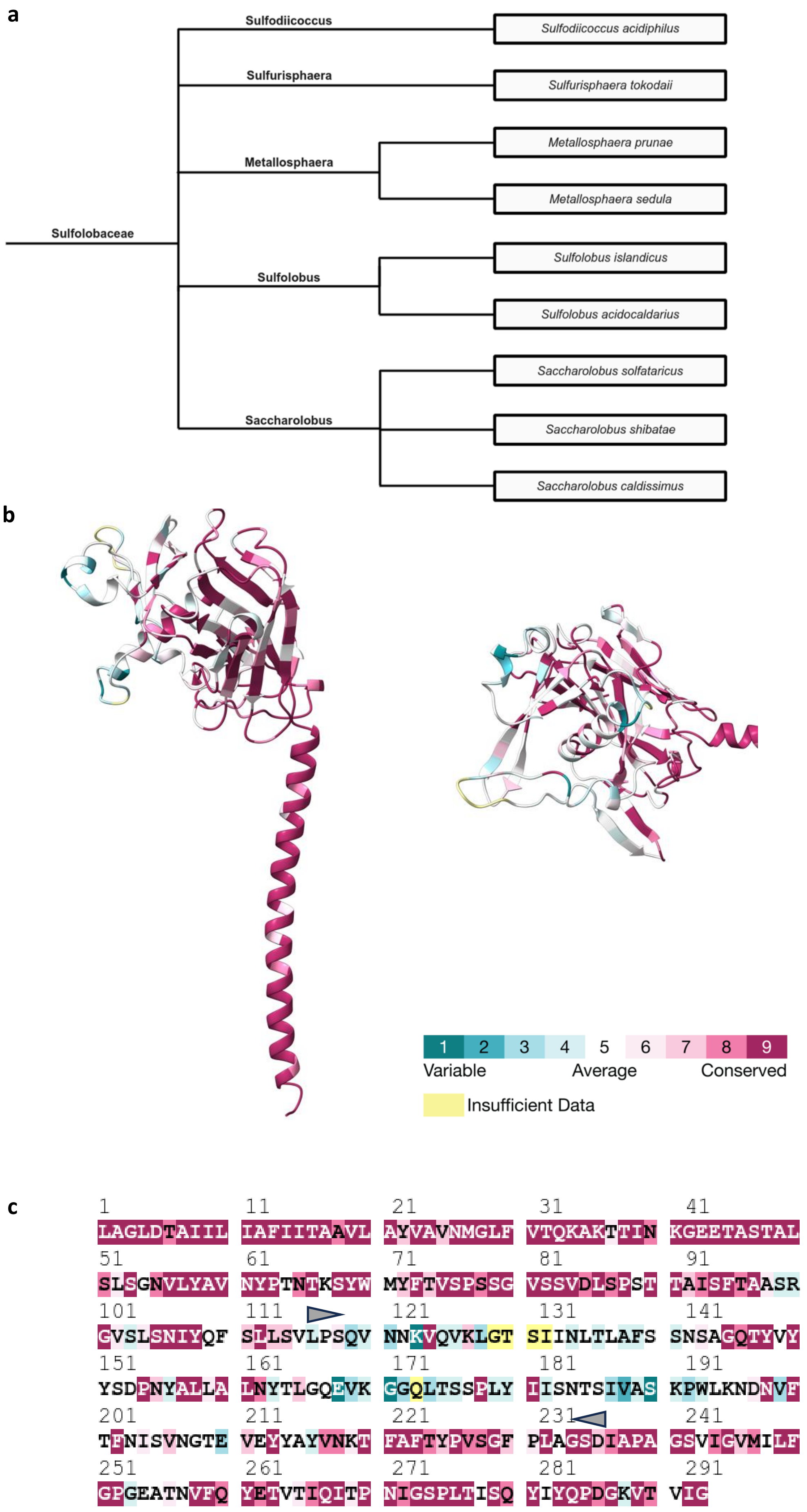

a
Sulfolobaceae
Sulfodiicoccus
Sulfurisphaera
Metallosphaera
Sulfolobus
Saccharolobus
Sulfodiicoccus acidiphilus
Sulfurisphaera tokodaii
Metallosphaera prunae
Metallosphaera sedula
Sulfolobus islandicus
Sulfolobus acidocaldarius
Saccharolobus solfataricus
Saccharolobus shibatae
Saccharolobus caldissimus
b
1 2 3 4 5 6 7 8 9
Variable
Average
Conserved
Insufficient Data
c
1 LAGLDTAIIL 11 IAFIITAAVL 21 AYVAVNMGLF 31 VTQKAKTTIN 41 KGEETASTAL
51 SLSGNVLYAV 61 NYPTNTKSYW 71 MYFTVSPSSG 81 VSSVDLSPST 91 TAISFTAASR
101 GVSLSNIYQF 111 SLLSVLPSQV 121 NNKVQVKLGT 131 SIINLTLAFS 141 SNSAGQTYVY
151 YSDPNYALLA 161 LNYTLGQEVK 171 GGQLTSSPLY 181 IISNTSIVAS 191 KPWLKNDNVF
201 TFNISVNGTE 211 VEYYAYVNKT 221 FAFTYPVSGF 231 PLAGSDIAPA 241 GSVIGVMILF
251 GPGEATNVFQ 261 YETVTIQITP 271 NIGSPLTISQ 281 YIYQPDGKVT 291 VIG

Supplementary Figure 9: Conservation of ArlB homologs across Sulfolobales

a, phylogenetic tree showing 9 Sulfolobales species sharing homologous ArlB proteins. **b,** ConSurf [4], shows the degree of conservation in ArlB. The ArlB structure is coloured by similarity, based on the species in a. **c**, amino acid sequence of *S. acidocaldarius* ArlB coloured by conservation. In b and c, regions of high conservation are maroon and regions of low conservation are cyan. Note that the glycosylation domain is the most divergent part of the protein. In c, the glycosylation domain is indicated by grey arrowheads.

```
HS1genome_1617_Sulfodiicoccus_acidiphilus   MSGRQLIIAVKRVSLKSAIRAYNRKMRRRGLAGLDTAIILIAFIITASVLAYVAVNMGLF 60
MsedA_1348_Metallosphaera                   -----------MGKIRGLVQKYNKLVRRRGLAGLDTAIILIAFIITASVLAFVAINMGLF 49
DFR88_11670_Metallosphaera_prunae           -----------MGKIRGLVQKYNKLVRRRGLAGLDAAIILIAFIITASVLAFVAINMGLF 49
Saci_1178_Sulfolobus_acidocaldarius         ------------------------------LAGLDTAIILIAFIITAAVLAYVAVNMGLF 30
SiRe_0124_Sulfolobus_islandicus             ------------MNTKKMLKEYNKKVKRKGLAGLDTAIILIAFIITASVLAYVAINMGLF 48
J5U21_01231_Saccharolobus_shibatae          ------------MGLQKIVKKYNKKMKRKGLAGLDTAIILIAFIITASVLAYVAINMGLF 48
SULC_0127_Saccharolobus_solfataricus        ------------MNSKKMLKEYNKKVKRKGLAGLDTAIILIAFIITASVLAYVAINMGLF 48
SACC_19750_Saccharolobus_caldissimus        ------------MGLKNAIKKYNKKVKRKGLAGLDTAIILIAFIITASVLAYVAINMGLF 48
STK_25180_Sulfurisphaera_tokodaii           ------------MGAKNAIKKYNKIVKRKGLAGLDTAIILIAFIITASVLAYVAINMGLF 48
                                                                          *****:***********:***:**:*****

HS1genome_1617_Sulfodiicoccus_acidiphilus   VTQKAKSTITKGEETASTALTLSGSVLYAVNYPTDTNSYWLYFPVSPSSGVSSVELSPAT 120
MsedA_1348_Metallosphaera                   VTQKAKTTINKGEETASTALTVSSSVLYAVNYPSNTKSYWIYFTVSPTSGVSSVELDPAT 109
DFR88_11670_Metallosphaera_prunae           VTQKAKTTINKGEETASTALTVSSSVLYAVNYPSNTKSYWIYFTVSPTSGVSSVELDPAT 109
Saci_1178_Sulfolobus_acidocaldarius         VTQKAKTTINKGEETASTALSLSGNVLYAVNYPTNTKSYWMYFTVSPSSGVSSVDLSPST 90
SiRe_0124_Sulfolobus_islandicus             VTQKAKSTINKGEETASTALTLSGSVLYAVNYPSNTRSYWIYFTVSPSSGVSSVELSPST 108
J5U21_01231_Saccharolobus_shibatae          VTQKAKSTINKGEETASTALTLSGSVLYAVNYPSNTRSYWIYFTVSPSSGVSSVELSPTT 108
SULC_0127_Saccharolobus_solfataricus        VTQKAKSTINKGEETASTALTLSGSVLYAVNYPLNTRSYWIYFTVSPSSGVSSVELSPTT 108
SACC_19750_Saccharolobus_caldissimus        VTQKAKSTINKGEETASTALTLSGSVLYAVNYPTNTRSYWIYFTVSPSSGVSSVELSPAT 108
STK_25180_Sulfurisphaera_tokodaii           VTQKAKSTINKGEETASTALTLSGSVLYAVNYPTNTKSYWIFFTVSPSSGVSSVELSPAT 108
                                            ******:**.**********::*..******** :*.***::* ***:******:*.*:*

HS1genome_1617_Sulfodiicoccus_acidiphilus   TAISFTASSSGIELSNIYVFSLMSYVSNY-------------YVTQTVSNQSQGGQTYYY 167
MsedA_1348_Metallosphaera                   TGLSFIATEEGITLSNIYKYTLLTDTHLTP---VS----ASGYSLTLESNLTSGGNTYYY 162
DFR88_11670_Metallosphaera_prunae           TGLSFIATEEGITLSNIYKYTLLTDTHLTP---VS----ASGYSLTLESNLTSGGNTYYY 162
Saci_1178_Sulfolobus_acidocaldarius         TAISFTAASRGVSLSNIYQFSLLSVLPSQVNNKVQVKLGTSIINLTLAFSSNSAGQTYVY 150
SiRe_0124_Sulfolobus_islandicus             TAISFTASAEGISYSNIYEYTLLTVSPSELANQVYANG----QYLDLVNQQTNAGQTYVY 164
J5U21_01231_Saccharolobus_shibatae          TAISFTASTEGIAYSNIYKYTLLTVDPSSLGNAVYANG----QYLNLINQQTSAGQTYVY 164
SULC_0127_Saccharolobus_solfataricus        TAISFTASAEGVTYSNIYKYTLLTVSPSELANVVYANG----QYLDLVNQQTSAGQTYVY 164
SACC_19750_Saccharolobus_caldissimus        TAISFTASSLGIAYSNIYKYTLLTVSPSEVNGIVYVNG----QYLNLADQETSGGQTYVY 164
STK_25180_Sulfurisphaera_tokodaii           TAISFTASTEGIAYSNIYKYTLLTVSPSTLSDVVYSNS----QYLSLVDTESSGGQTYVY 164
                                            *.:** *:  *:  **** ::*::                           ...*:** *

HS1genome_1617_Sulfodiicoccus_acidiphilus   FTSPYAALVALSEIISNKQNT-------TPVPVNISTT--------KPTTTTDVNTFSFE 212
MsedA_1348_Metallosphaera                   FSSPYLALLALNQSLSKV---------SGHSPIYINYTS-FSSTNPEPSWLKNDNNFTFT 212
DFR88_11670_Metallosphaera_prunae           FSSPYLALLALNQSLSKV---------SGHSPIYINYTS-FSSTNPEPSWLKNDNNFTFT 212
Saci_1178_Sulfolobus_acidocaldarius         YSDPNYALLALNYTLGQEVKGGQ----LTSSPLYIISNT---SIVASKPWLKNDNVFTFN 203
SiRe_0124_Sulfolobus_islandicus             YPNPYYALLALNYTLSKI--DK-----VSPSPLYITTTT-PSSATQIYPFLAHDNMFTFT 216
J5U21_01231_Saccharolobus_shibatae          YPNPYYALLALNYTLYNYYLKT-----KTPSPIFINSST----LSSIPQWLKNDNSFTFT 215
SULC_0127_Saccharolobus_solfataricus        YPNPYYALLALNYTLYNYYLST-----KTPSPIFINSSI-L-SLSSLPSWLKNDNSFTFT 217
SACC_19750_Saccharolobus_caldissimus        YPNPYYALLALNYTLGQLYKPSTYSTTKASTPLYIATSTQVSTLLTTMPWLKNDNVFSFT 224
STK_25180_Sulfurisphaera_tokodaii           YPNPYYALLALNYTLSQKVNDKII----KYSPLFIN----YTPIPTKPSWLQSDNVFSFT 216
                                            : .*  **:**.  : :              *: *                   * *:*

HS1genome_1617_Sulfodiicoccus_acidiphilus   ----YQRTTYYVTVYRTFAFTFPIAGFSLAGSDVAPAGSTIGVMVLFGPHAGSQVFQYQT 268
MsedA_1348_Metallosphaera                   LTIAGQKVLYYVFINQTFAFSYPVAGDPLVGSAIAPAGSTVGFMILFGPNLGQHVFEYQT 272
DFR88_11670_Metallosphaera_prunae           LTIAGQKVLYYVFINQTFAFSYPVAGDPLVGSAIAPAGSTVGFMILFGPNLGQHVFEYQT 272
Saci_1178_Sulfolobus_acidocaldarius         ISVNGTEVEYYAYVNKTFAFTYPVSGFPLAGSDIAPAGSVIGVMILFGPGEATNVFQYET 263
SiRe_0124_Sulfolobus_islandicus             LNISGTLVTYYAFVNQTFAFTYPVAGDPLIGSAIAPAGSVIGVMILFGPDLGSHVFQYQT 276
J5U21_01231_Saccharolobus_shibatae          LNISGQLVTYDVFVNQTFAFTYPVAGDPLIGSAIAPAGSVIGVMILFGPDLGSHVFQYQT 275
SULC_0127_Saccharolobus_solfataricus        LNISGKLVTYYVFVNQTFAFTYPVAGDPLIGSAIAPAGSVIGVILLFGPDLGSHVFQYQT 277
SACC_19750_Saccharolobus_caldissimus        LNISGQLVTYYAYVNQTFAFTYPVAGDPLIGSAIAPAGSVIGVMLLFGPDLGSHVFQYQT 284
STK_25180_Sulfurisphaera_tokodaii           LNISGKPVTYCAFVNQTFAFTYPVAGDPLVGSAIAPAGSVIGVMILFGPSLGSHVFQYQT 276
                                                   . * . : :****::*::*  * ** :*****.:*.::****  . :**:*:*

HS1genome_1617_Sulfodiicoccus_acidiphilus   VTVQITPNIGSPLTVSEYIYQPEGNVTIIG 298
MsedA_1348_Metallosphaera                   INIQITPNIGSPLTLSEYIYQPEGTVTAIG 302
DFR88_11670_Metallosphaera_prunae           INIQITPNIGSPLTLSEYIYQPEGTVTAIG 302
Saci_1178_Sulfolobus_acidocaldarius         VTIQITPNIGSPLTISQYIYQPDGKVTVIG 293
SiRe_0124_Sulfolobus_islandicus             ITIQITPNIGSPLTISEYVYQPEGSVSVIG 306
J5U21_01231_Saccharolobus_shibatae          VTIQITPNVGSPLTISEYVYQPEGSVSVIG 305
SULC_0127_Saccharolobus_solfataricus        ITIQITPNIGSPLTISEYIYQPEGSVSVIG 307
SACC_19750_Saccharolobus_caldissimus        ITIQISPNIGSPLTLSEYVYQPEGNVTVIG 314
STK_25180_Sulfurisphaera_tokodaii           ISIQISPNIGSPLTLSEYIYQPEGTVTVIG 306
                                            :.:**:**:*****:*:*:***:*.*: **
```

Supplementary Figure 10: ArlB homologs in sulfolobales

Multisequence alignment of archaellins from 9 sulfolobales species (as in Supplementary Figure 9). Sequences highlighted in yellow show full residue conservation amongst all 9 species, red amino acids are glycosylation sites, magenta boxes show residues overlapping with metal binding sites in euryarchaeota. Note that the analogous residues in crenarchaeota are not capable of coordinating metals. Grey arrowheads with interconnecting dashed lines delineate the glycosylation domain in *S. acidocaldarius.*

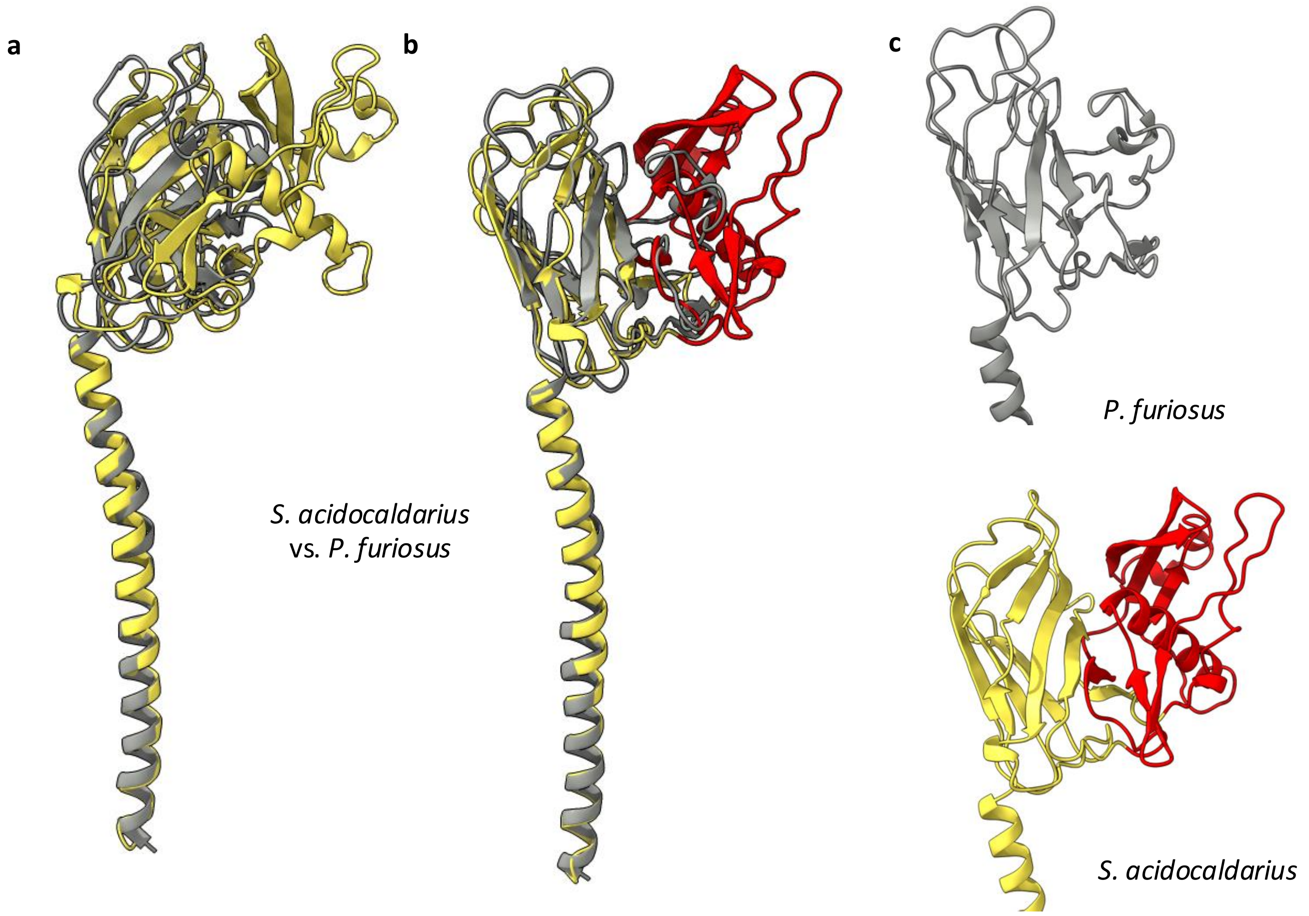
a
b
c
S. acidocaldarius
vs. P. furiosus
P. furiosus
S. acidocaldarius

Supplementary Figure 11: Glycosylation subdomain in crenarchaeota

a, superimposition of the archaellin ArlB from *S. acidocaldarius* (yellow) with FlaB0 from *P. furiosus* [5] (PDB-5O4U; grey). **b,** highlights the glycosylation sub-domain of *S. acidocaldarius* ArlB in red. **c,** archaellin head domains of *P. furiosus* and *S. acidocaldarius* side-by-side.

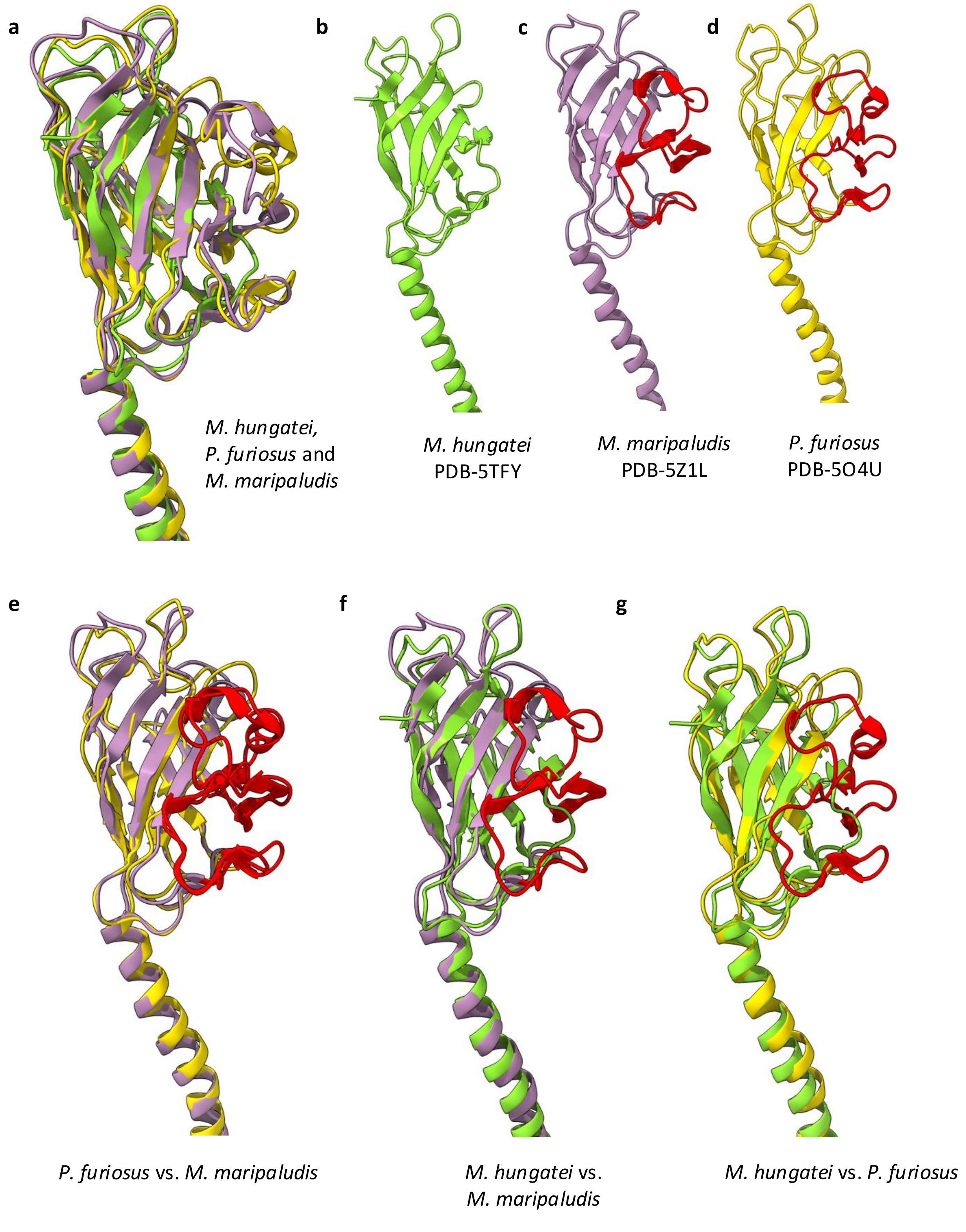
a
b
c
d
M. hungatei,
P. furiosus and
M. maripaludis
M. hungatei
PDB-5TFY
M. maripaludis
PDB-5Z1L
P. furiosus
PDB-5O4U
e
f
g
P. furiosus vs. *M. maripaludis*
M. hungatei vs.
M. maripaludis
M. hungatei vs. *P. furiosus*

Supplementary Figure 12: Structural divergence of archaellins in euryarchaea

a, superimposition of the archaellins of *M. hungatei* (PDB-5TFY[6]; green), *M. maripaludis* (PDB-5Z1L[7], purple) and and *P. furiosus* (PDB-5O4U[5], yellow). **b**-**d,** archaellins from (a) shown side-by-side. Red backbone sections visualise areas of structural variability. **e-g**, pairwise superimposition *of P. furiosus* and *M. maripaludis* (**e**); *M. hungatei* and *M. maripaludis* (**f**); and *M. hungatei* vs. *P. furiosus* (**g**).

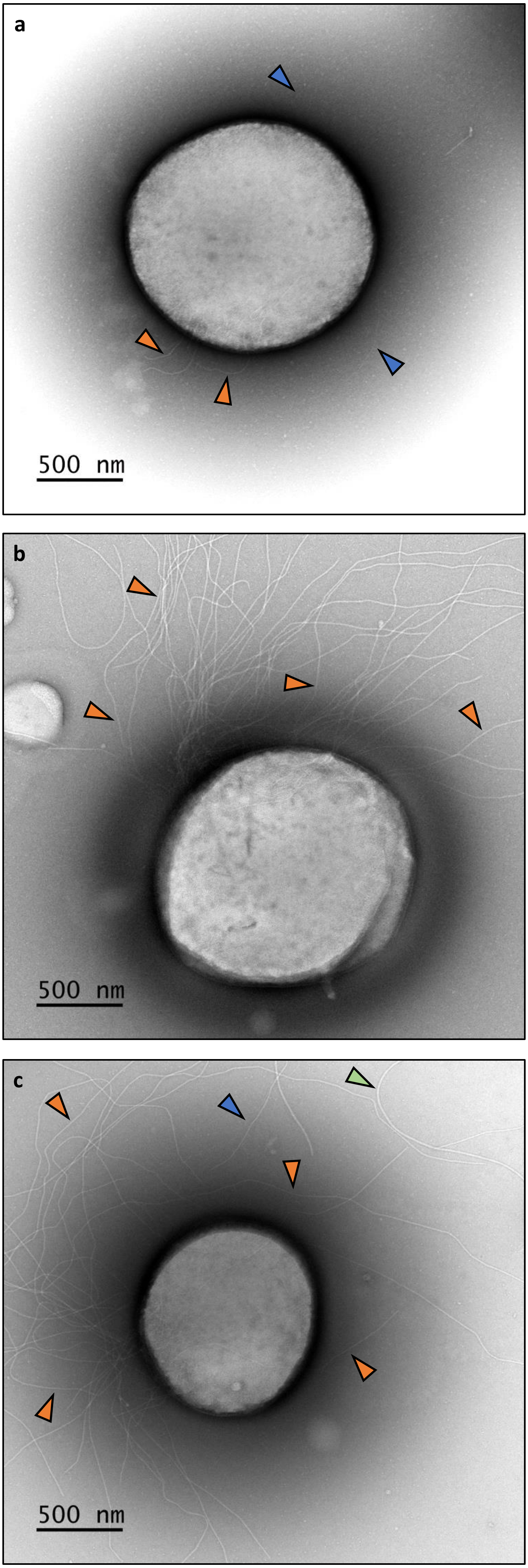
a
500 nm
b
500 nm
c
500 nm

Supplementary Figure 13: Growth-phase dependent expression of filaments

S. acidocaldarius DSM639 at (**a**) early lag; (**b**) late logarithmic; and (**c**) stationary growth phase. Cells in early lag phase produce mainly threads (blue arrowheads). Aap (orange arrowheads) are produced soon after - from late log to stationary phase. Archaella (green arrowheads) appear mostly in stationary phase with 0-5 archaella per cell.

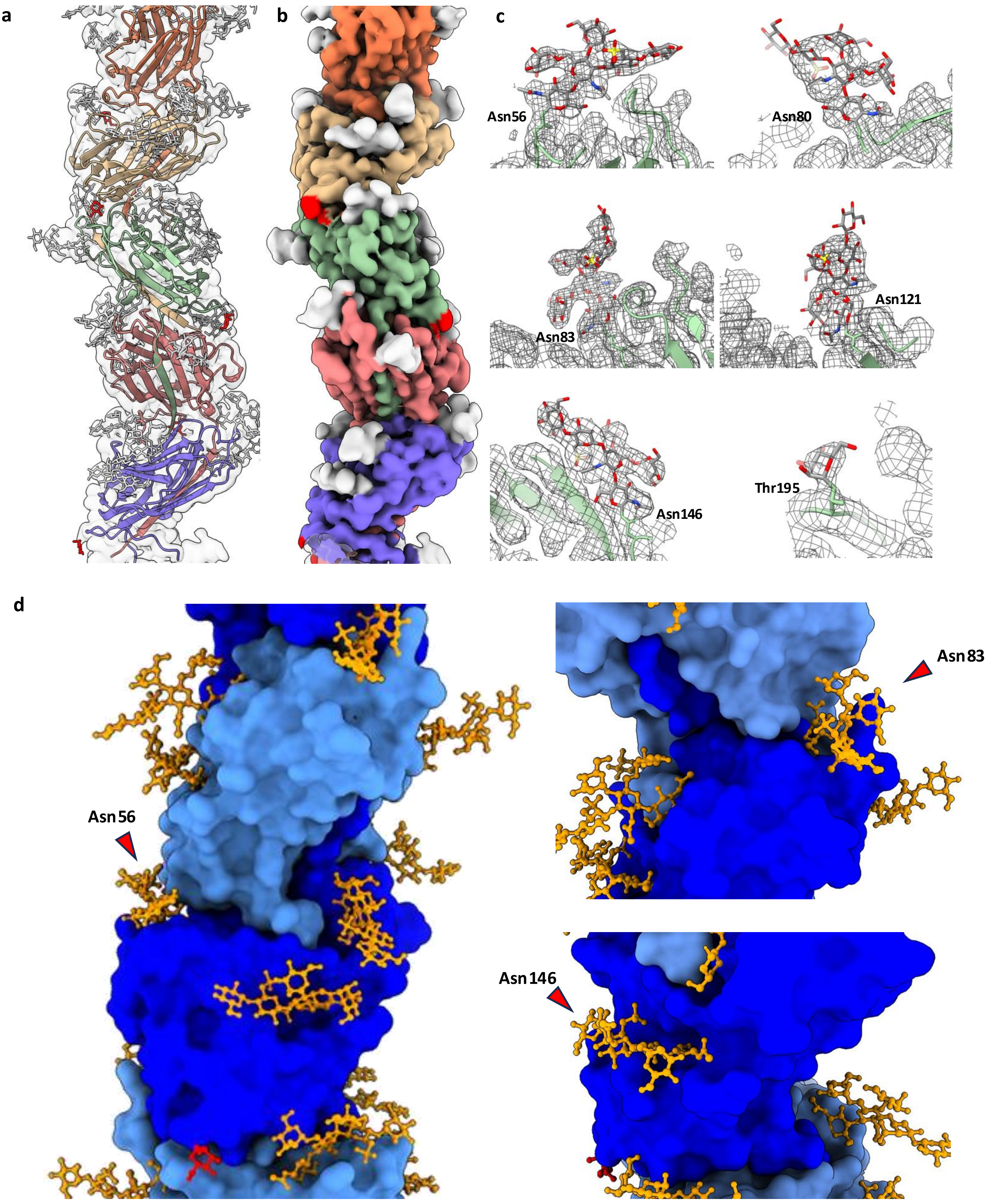

a
b
c
Asn56
Asn80
Asn83
Asn121
Asn146
Thr195
d
Asn56
Asn83
Asn146

Supplementary Figure 14: 2.7 Å resolution cryoEM map and model of the thread with full length glycans

a, map of the *S. acidocaldarius* thread at 2.7 Å resolution (transparent grey) and atomic model in ribbon representation (multicolour). N-glycans are white sticks, O-glycans are red sticks. **b**, cryoEM map of the thread coloured according to the model in a. A high contour level was set to visualize the weaker glycan densities. **c**, closeups of the five N-glycans (bound to Ans56, Ans80, Ans83, Ans121 and Ans146), and one O-glycan (bound to Thr195) of the thread subunit Saci_0406. Protein is shown as green ribbons and glycans are coloured by element. **d**, closeups showing how glycans in the thread are wedged into clefts between subunits (Asn56 and Asn83) or within the same subunit (Asn146). Protein is shown as surface (alternating subunits are couloured mid and light blue). N-glycans as orange balls and sticks and O-glycans red balls and sticks.

S. acidocaldarius wt* (MW001)** ***S. acidocaldarius Δagl3

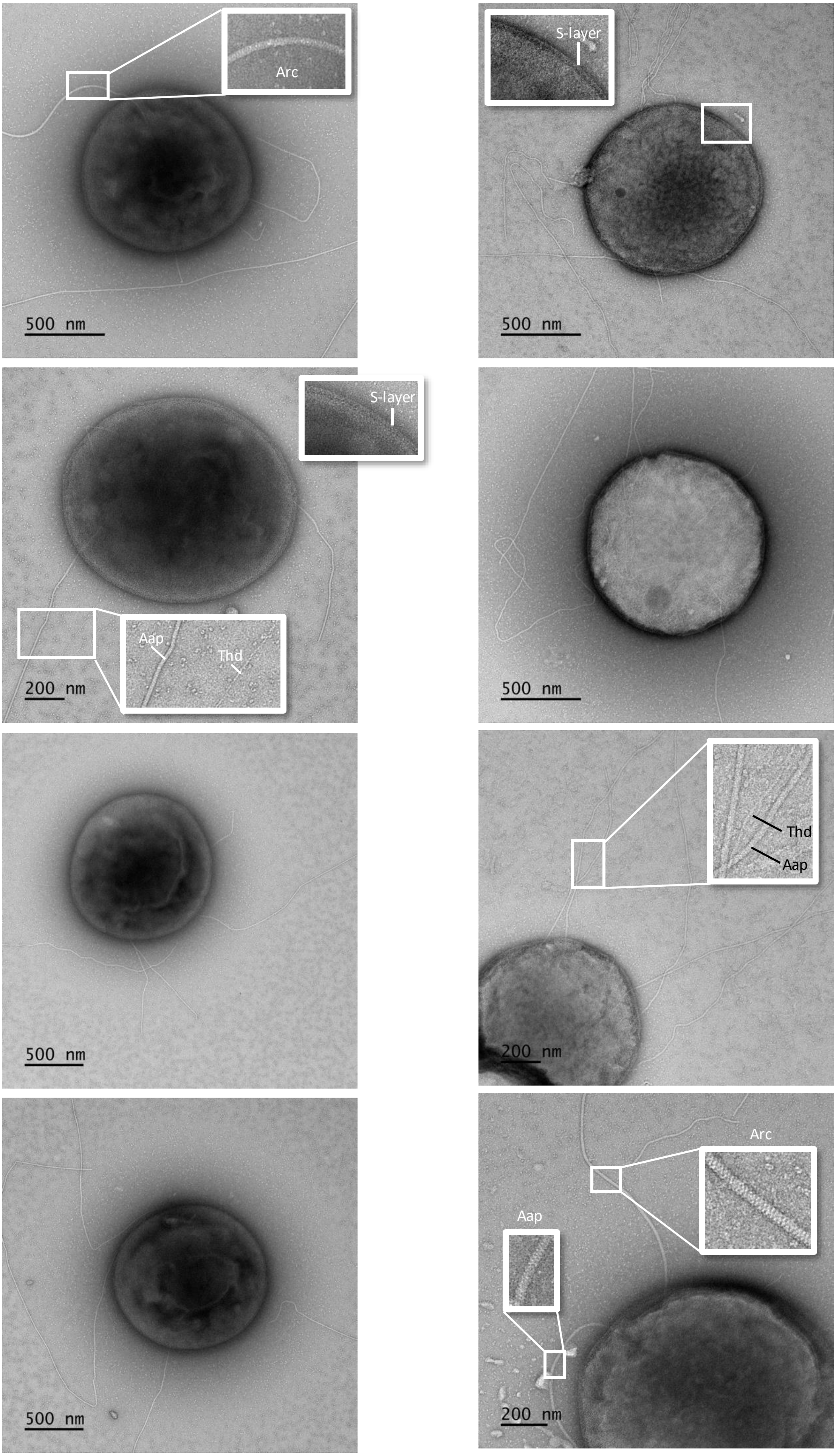

Supplementary Figure 15: Morphology of the Δagl3 mutant

Negative stain images of *S. acidocaldarius wt* (MW001) (left panel) and the Δagl3 mutant cells (right panel) do not show obviously distinct phenotypes. Arc, archaellum; Thd, thread.

***S. acidocaldarius wt* (MW001)** ***S. acidocaldarius Δagl3* (MW039)**

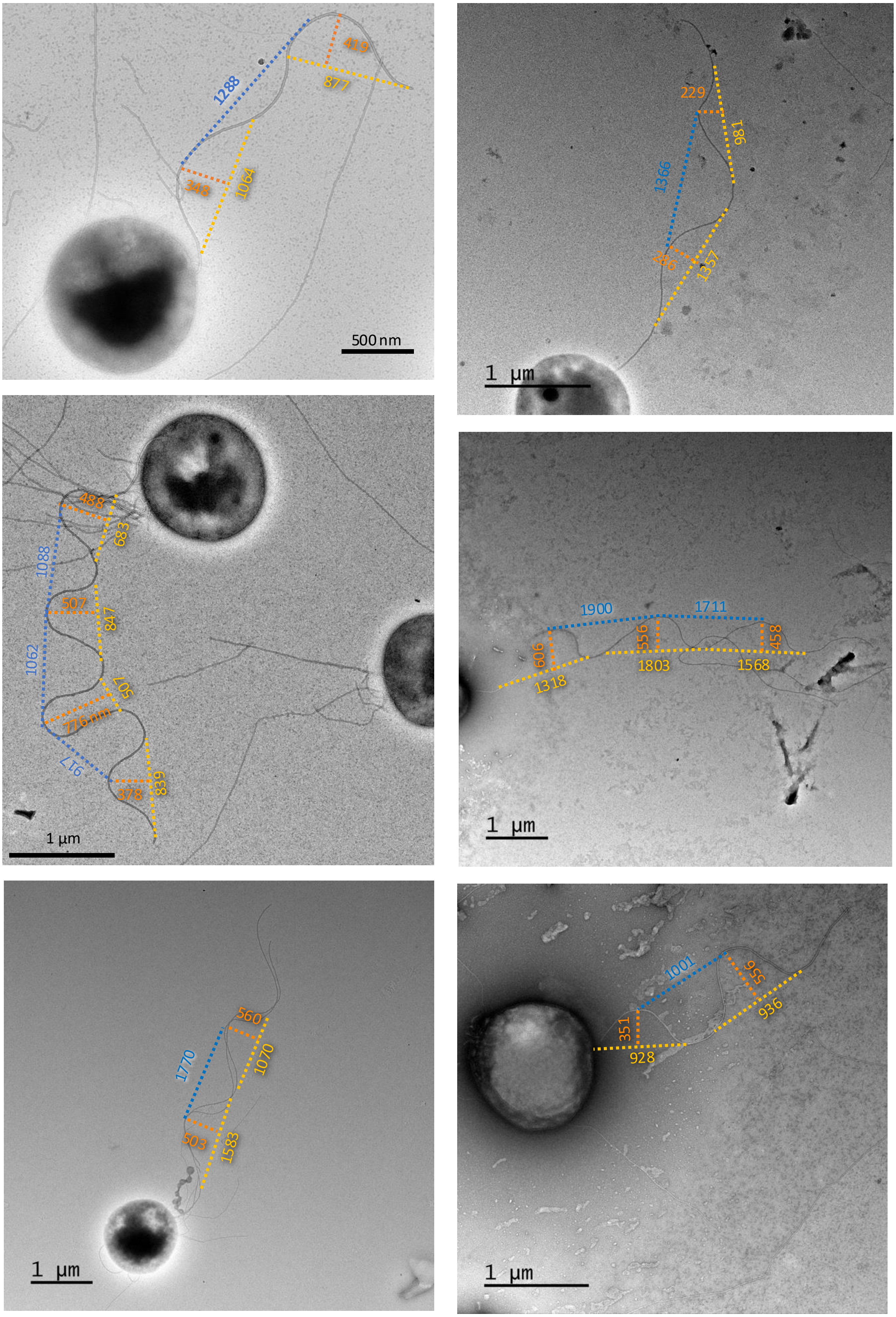

Supplementary Figure 16: Archaella of the wt and Δagl3 mutant in comparison

Negative stain images of *S. acidocaldarius wt* (MW001) (left panel) and the Δagl3 mutant cells (MW039) (right panel) displaying the whole lengths of their archaella. The apparent lengths and waveforms of the archaella vary significantly within the same sample. Therefore, clear conclusions about differences in these parameters between WT and the mutant cells cannot be drawn. All measurements in nm.

Supplementary Table 1 - Cryo-EM data collection, refinement and validation statistics for *S. acidocaldarius* wild type filaments with full-length glycans

	wt archaellum (EMDB-18700) (PDB 8QX4)	wt thread (EMDB-19608) (PDB 8RZL)	wt Aap (EMDB-19960) (PDB 9ETS)
**Data collection and processing**			
Magnification	105 k	105 k	105 k
Voltage (kV)	300	300	300
Electron exposure (e–/Å^2)	43.27	43.27	40
Defocus range (μm)	-1.0 to -2.5	-1.0 to -2.5	-1.0 to -2.1
Pixel size (Å)	0.829	0.829	1.07
Symmetry imposed	Twist: 107.923°<br>Rise: 5.43 Å	Twist: -103.281°<br>Rise: 31.650 Å	Twist: -39.859°<br>Rise: 15.524 Å
Initial particle images (no.)	1,705,100	2,852,968	7,833,953
Final particle images (no.)	1,059,736	626,078	505,862
Map resolution (Å)<br>FSC threshold	2.0<br>0.143	2.7<br>0.143	2.6<br>0.143
Map resolution range (Å)	1.8 – 2.3	2.5-3.0	2.3-2.8
Refinement			
Initial model used (PDB code)	Ab initio	7PNB	8Q30
Map/Model resolution (Å)<br>FSC threshold	2.2<br>0.5	2.8<br>0.5	2.8<br>0.5
Map sharpening B factor (Å^2)	n/a	n/a	n/a
Model composition<br>Non-hydrogen atoms<br>Protein residues<br>Ligands (glycans)	 51,960<br>5,860 (293x20)<br>620 (31x20)	 8,895<br>915 (183x5)<br>155 (31x5)	 42.941<br>5,217 (141x37)<br>456
B factors (Å^2)<br>Protein<br>Ligand	 77.8<br>159.5	 89.3<br>171.3	 91.95<br>224.9
R.m.s. deviations<br>Bond lengths (Å)<br>Bond angles (°)	 0.008<br>1.493	 0.008<br>1.498	 0.008<br>1.390
Validation<br>MolProbity score<br>Clashscore<br>Poor rotamers (%)	 1.76<br>3.44<br>0.40	 1.69<br>1.93<br>3.21	 1.06<br>2.15<br>1.23
Ramachandran plot<br>Favored (%)<br>Allowed (%)<br>Disallowed (%)	 97.94<br>2.06<br>0.0	 95.3<br>4.97<br>0.0	 99.05<br>0.95<br>0.0

Supplementary Table 2 - Cryo-EM data collection, refinement and validation statistics *for S. acidocaldarius* Δagl3 filaments with truncated glycans

	Δagl3 Archaellum (EMDB-18700) (PDB 8QX4)	Δagl3 Aap (EMDB-19608) (PDB 8RZL)
**Data collection and processing**		
Magnification	130 k	130 k
Voltage (kV)	300	300
Electron exposure (e–/Å^2)	40	40
Defocus range (μm)	-1.8 to -2.2	-0.8 to -2.2
Pixel size (Å)	0.829	0.829
Symmetry imposed	Twist: 107.923°<br>Rise: 5.399 Å	Twist: -103.281°<br>Rise: 15.282 Å
Initial particle images (no.)	1,174,572	3,560,700
Final particle images (no.)	256,869	691,479
Map resolution (Å)<br>FSC threshold	2.4<br>0.143	2.4<br>0.143
Map resolution range (Å)	2.2 – 2.7	2.5-3.0
Refinement		
Initial model used (PDB code)	8QX4	8Q30
Map/Model resolution (Å)<br>FSC threshold	2.6<br>0.5	2.5<br>0.5
Map sharpening B factor (Å^2)	n/a	n/a
Model composition<br>Non-hydrogen atoms<br>Protein residues<br>Ligands (glycans)	 48,800<br>5,860 (293x20)<br>360 (18x20)	 33,540<br>4230 (141x30)<br>260 (9x30)
B factors (Å^2)<br>Protein<br>Ligand	 81.1<br>170.9	 61.4<br>131.4
R.m.s. deviations<br>Bond lengths (Å)<br>Bond angles (°)	 0.008<br>1.540	 0.008<br>1.596
Validation<br>MolProbity score<br>Clashscore<br>Poor rotamers (%)	 1.07<br>2.31<br>1.21	 1.28<br>5.26<br>0.30
Ramachandran plot<br>Favored (%)<br>Allowed (%)<br>Disallowed (%)	 98.26<br>1.74<br>0.0	 99.52<br>0.48<br>0.0

Supplementary References

1. Nuno de Sousa Machado J, Albers SV, Daum B. Towards Elucidating the Rotary Mechanism of the Archaellum Machinery. Front Microbiol. 2022 Mar 21;13:848597. doi: 10.3389/fmicb.2022.848597
2. Teufel F, Almagro Armenteros JJ, Johansen AR, et al. SignalP 6.0 predicts all five types of signal peptides using protein language models. Nature Biotechnology 2022 40:7. 2022;40(7):1023-1025. doi:10.1038/s41587-021-01156-3
3. Kreutzberger MAB, Sonani RR, Liu J, et al. Convergent evolution in the supercoiling of prokaryotic flagellar filaments. *Cell*. 2022;185(19):3487-3500.e14. doi:10.1016/J.CELL.2022.08.009
4. Ashkenazy H, Abadi S, Martz E, et al. ConSurf 2016: an improved methodology to estimate and visualize evolutionary conservation in macromolecules. Nucleic Acids Res. 2016;44(W1):W344-W350. doi:10.1093/NAR/GKW408
5. Daum B, Vonck J, Bellack A, et al. Structure and in situ organisation of the pyrococcus furiosus archaellum machinery. *Elife*. 2017;6. doi:10.7554/ELIFE.27470
6. Poweleit N, Ge P, Nguyen HH, Loo RRO, Gunsalus RP, Zhou ZH. CryoEM structure of the Methanospirillum hungatei archaellum reveals structural features distinct from the bacterial flagellum and type IV pilus. *Nature Microbiology 2016 2:3*. 2016;2(3):1-12. doi:10.1038/nmicrobiol.2016.222
7. Meshcheryakov VA, Shibata S, Schreiber MT, et al. High-resolution archaellum structure reveals a conserved metal-binding site. *EMBO Rep*. 2019;20(5):e46340. doi:10.15252/EMBR.201846340
