# Supplementary material for: Towards a molecular picture of the archaeal cell surface
Source: Nat Commun. 2024 Nov 29;15:10401. doi: 10.1038/s41467-024-53986-9 (PMC11607397; doi:10.1038/s41467-024-53986-9)
Supplement: Supplementary file 2 — Description of Additional Supplementary Files [file 41467_2024_53986_MOESM2_ESM.pdf]

Description of Additional Supplementary Files:

Supplementary Data 1: This zip file contains all Alphafold predictions and metadata of archaellin homologs from related Sulfolobales species presented in this manuscript.

Supplementary Movie 1: Maps and models of wild type and Δagl3 archaella, Aap, and threads. Glycans have been omitted for clarity.

Supplementary Movie 2: Maps and models of wild type and Δagl3 archaella, Aap, and threads, focusing on the glycans.
