# Supplementary figures and images for: Towards a molecular picture of the archaeal cell surface
Source: Nat Commun. 2024 Nov 29;15:10401. doi: 10.1038/s41467-024-53986-9 (PMC11607397; doi:10.1038/s41467-024-53986-9)

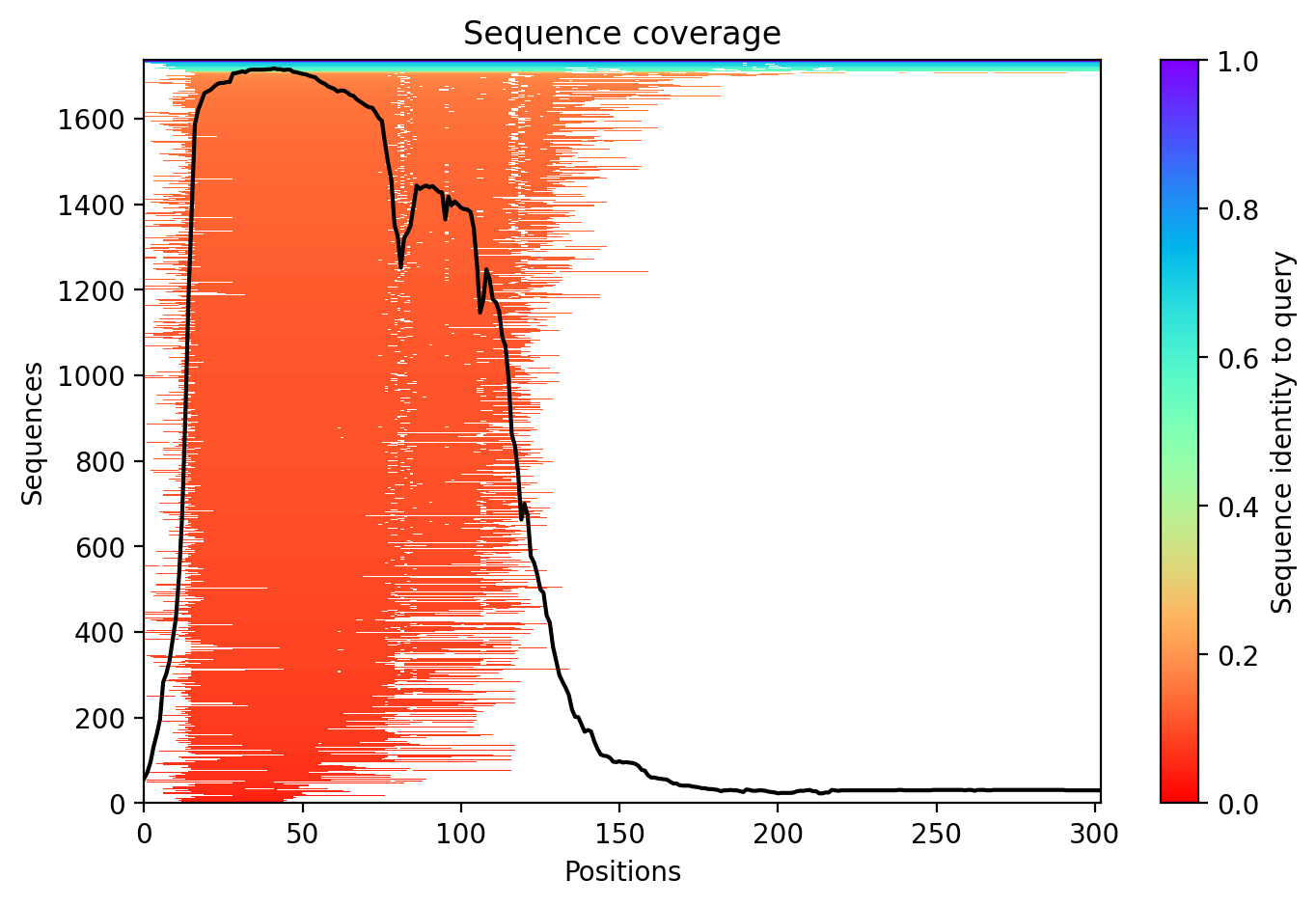

Supplement: Supplementary file 5 — Supplementary Dataset 1 [file 41467_2024_53986_MOESM5_ESM.zip › Archaellum_Homologues_2024/DFR88_11670_Metallosphaera_prunae/DFR88_11670_Metallosphaera_prunae_bda9b_coverage.png]

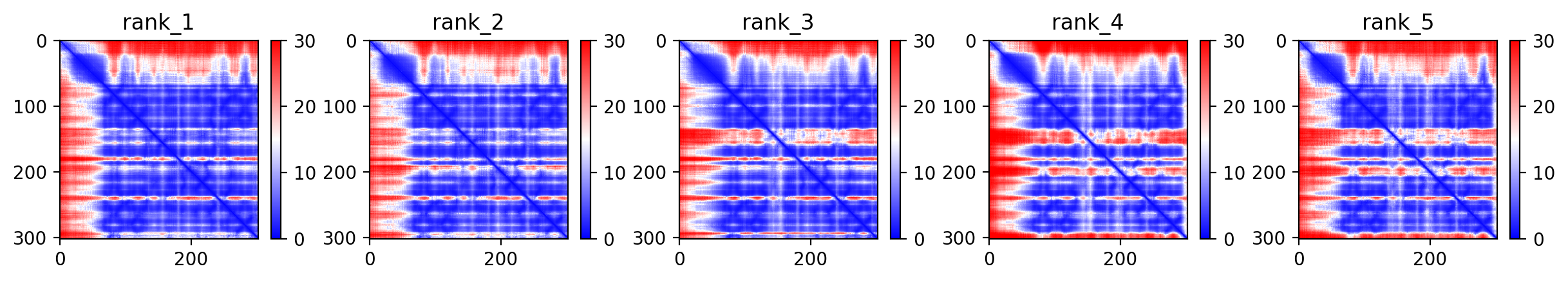

Supplement: Supplementary file 5 — Supplementary Dataset 1 [file 41467_2024_53986_MOESM5_ESM.zip › Archaellum_Homologues_2024/DFR88_11670_Metallosphaera_prunae/DFR88_11670_Metallosphaera_prunae_bda9b_pae.png]

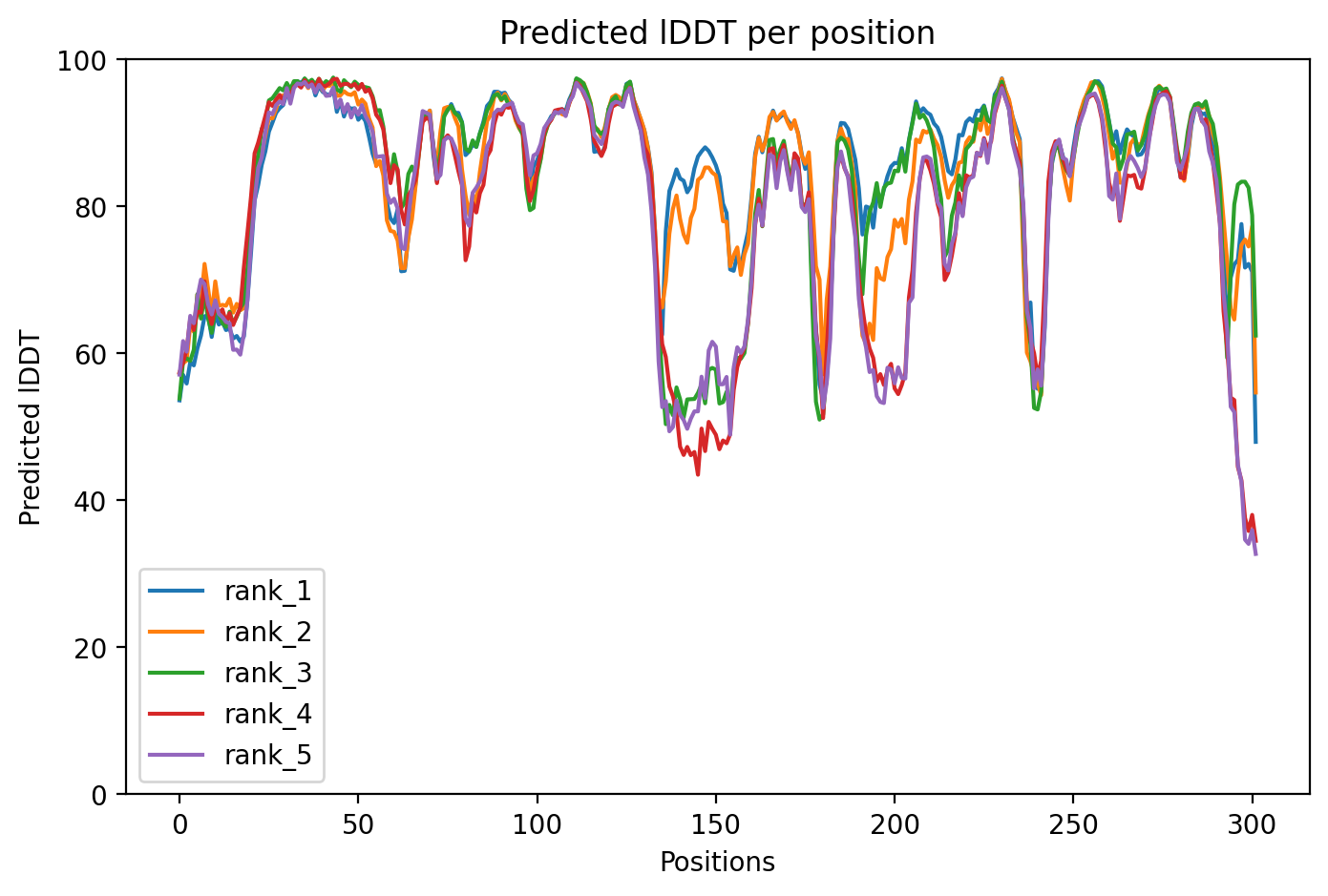

Supplement: Supplementary file 5 — Supplementary Dataset 1 [file 41467_2024_53986_MOESM5_ESM.zip › Archaellum_Homologues_2024/DFR88_11670_Metallosphaera_prunae/DFR88_11670_Metallosphaera_prunae_bda9b_plddt.png]

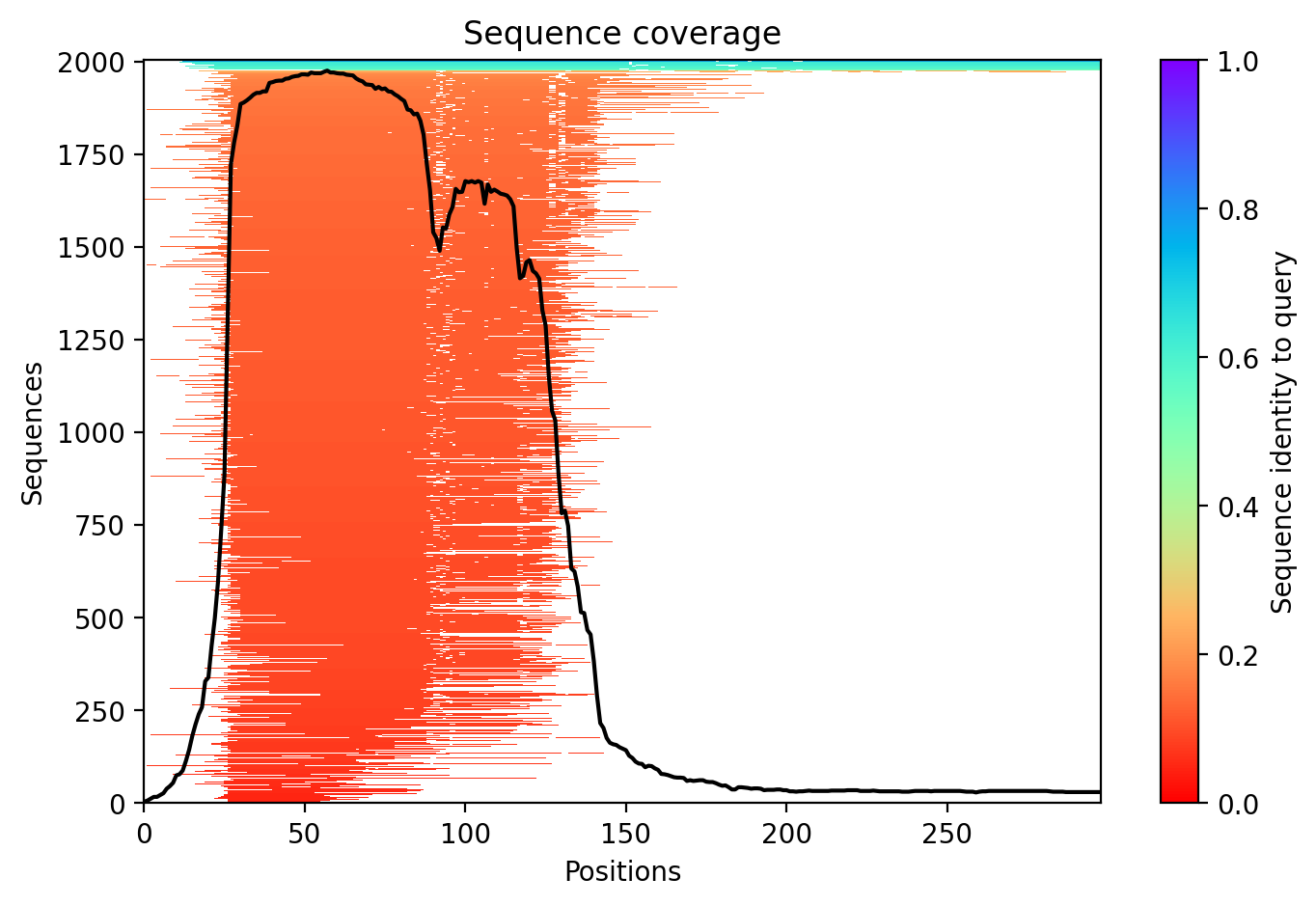

Supplement: Supplementary file 5 — Supplementary Dataset 1 [file 41467_2024_53986_MOESM5_ESM.zip › Archaellum_Homologues_2024/HS1genome_1617_Sulfodiicoccus_acidiphilus/HS1genome_1617_Sulfodiicoccus_acidiphilus_34e1f_coverage.png]

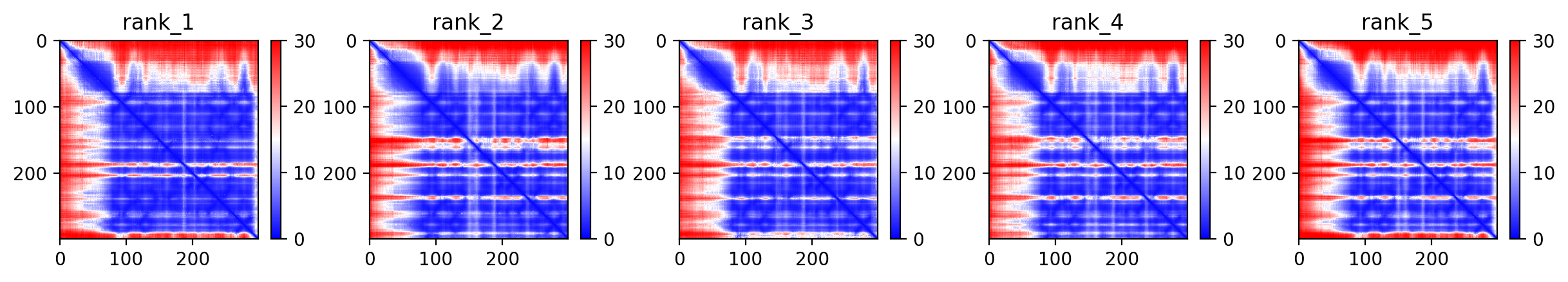

Supplement: Supplementary file 5 — Supplementary Dataset 1 [file 41467_2024_53986_MOESM5_ESM.zip › Archaellum_Homologues_2024/HS1genome_1617_Sulfodiicoccus_acidiphilus/HS1genome_1617_Sulfodiicoccus_acidiphilus_34e1f_pae.png]

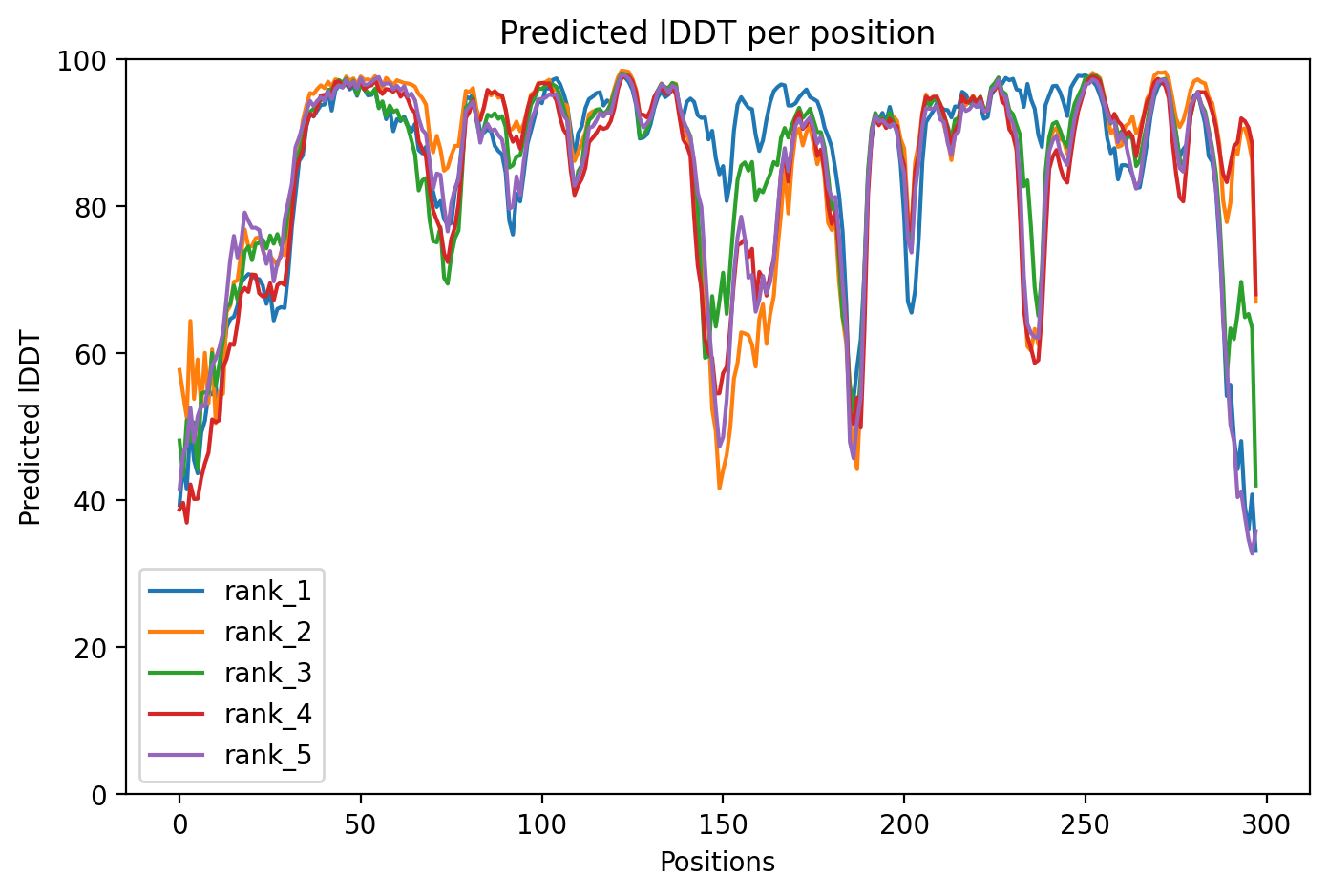

Supplement: Supplementary file 5 — Supplementary Dataset 1 [file 41467_2024_53986_MOESM5_ESM.zip › Archaellum_Homologues_2024/HS1genome_1617_Sulfodiicoccus_acidiphilus/HS1genome_1617_Sulfodiicoccus_acidiphilus_34e1f_plddt.png]

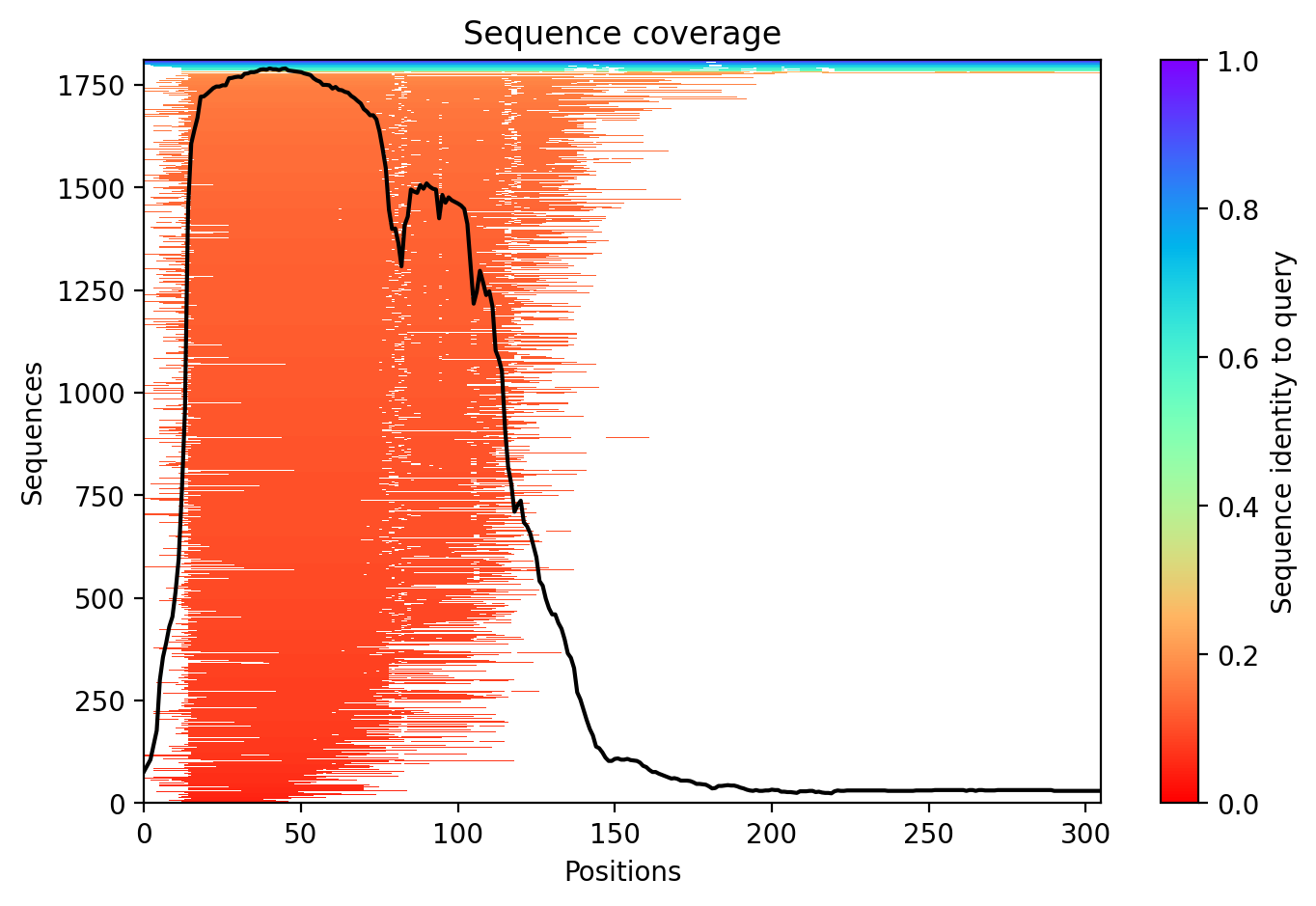

Supplement: Supplementary file 5 — Supplementary Dataset 1 [file 41467_2024_53986_MOESM5_ESM.zip › Archaellum_Homologues_2024/J5U21_01231_Saccharolobus_shibatae/J5U21_01231_Saccharolobus_shibatae_8965b_coverage.png]

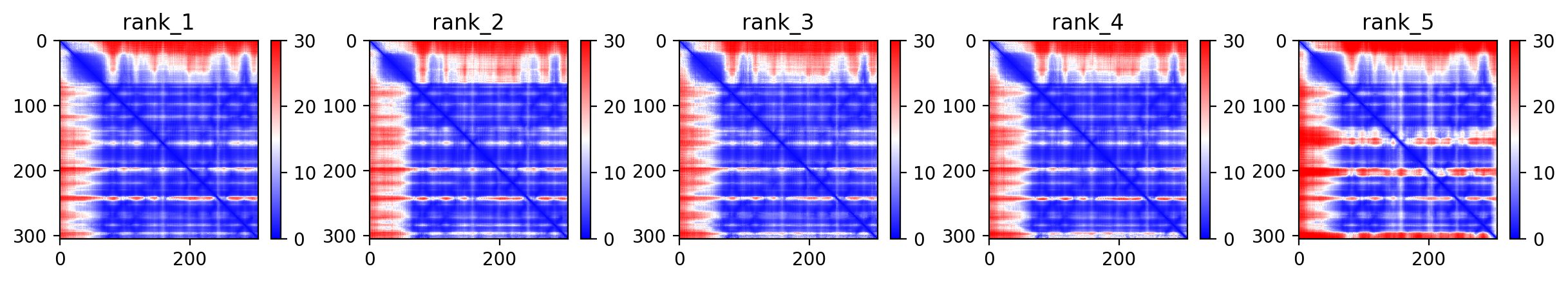

Supplement: Supplementary file 5 — Supplementary Dataset 1 [file 41467_2024_53986_MOESM5_ESM.zip › Archaellum_Homologues_2024/J5U21_01231_Saccharolobus_shibatae/J5U21_01231_Saccharolobus_shibatae_8965b_pae.png]

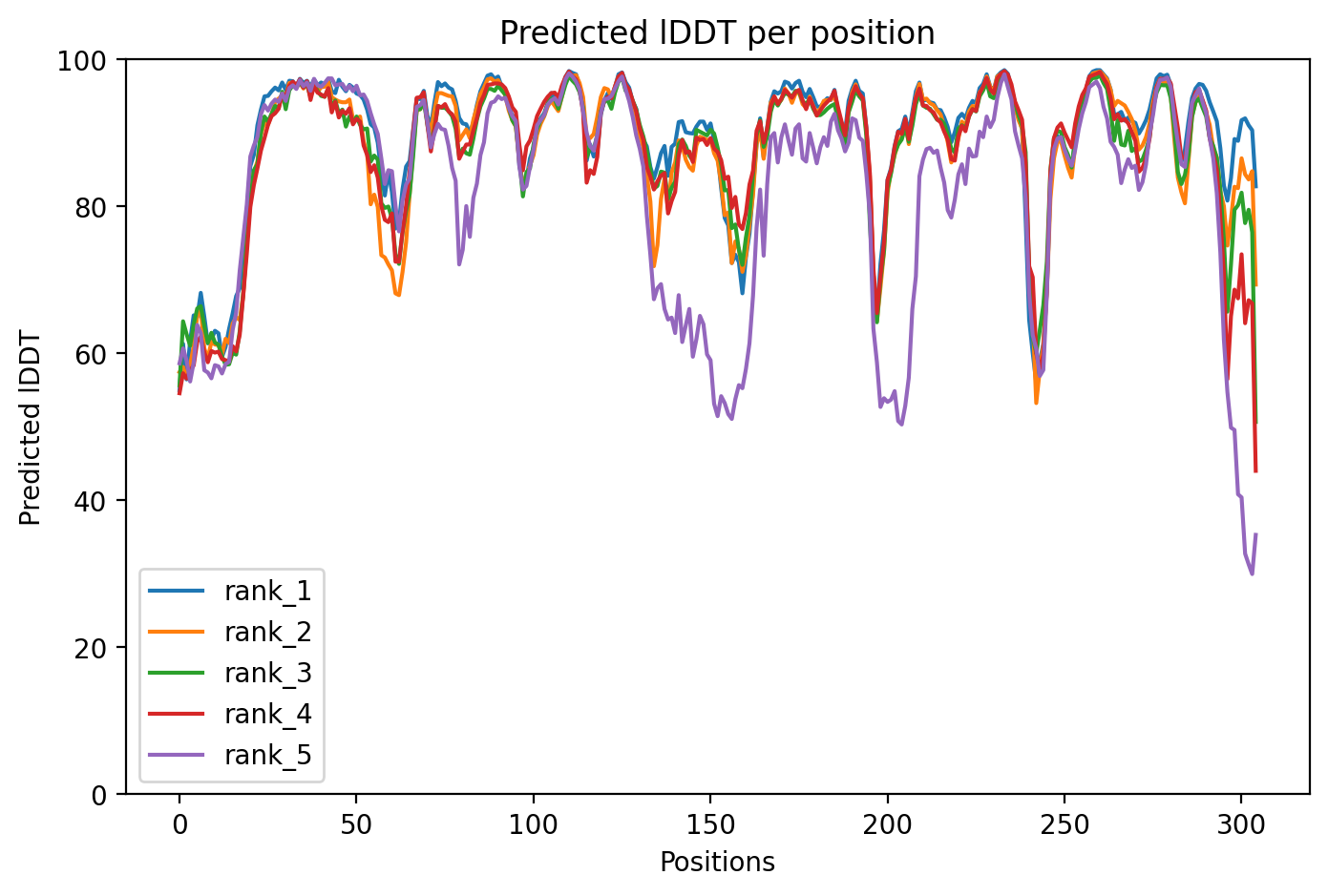

Supplement: Supplementary file 5 — Supplementary Dataset 1 [file 41467_2024_53986_MOESM5_ESM.zip › Archaellum_Homologues_2024/J5U21_01231_Saccharolobus_shibatae/J5U21_01231_Saccharolobus_shibatae_8965b_plddt.png]

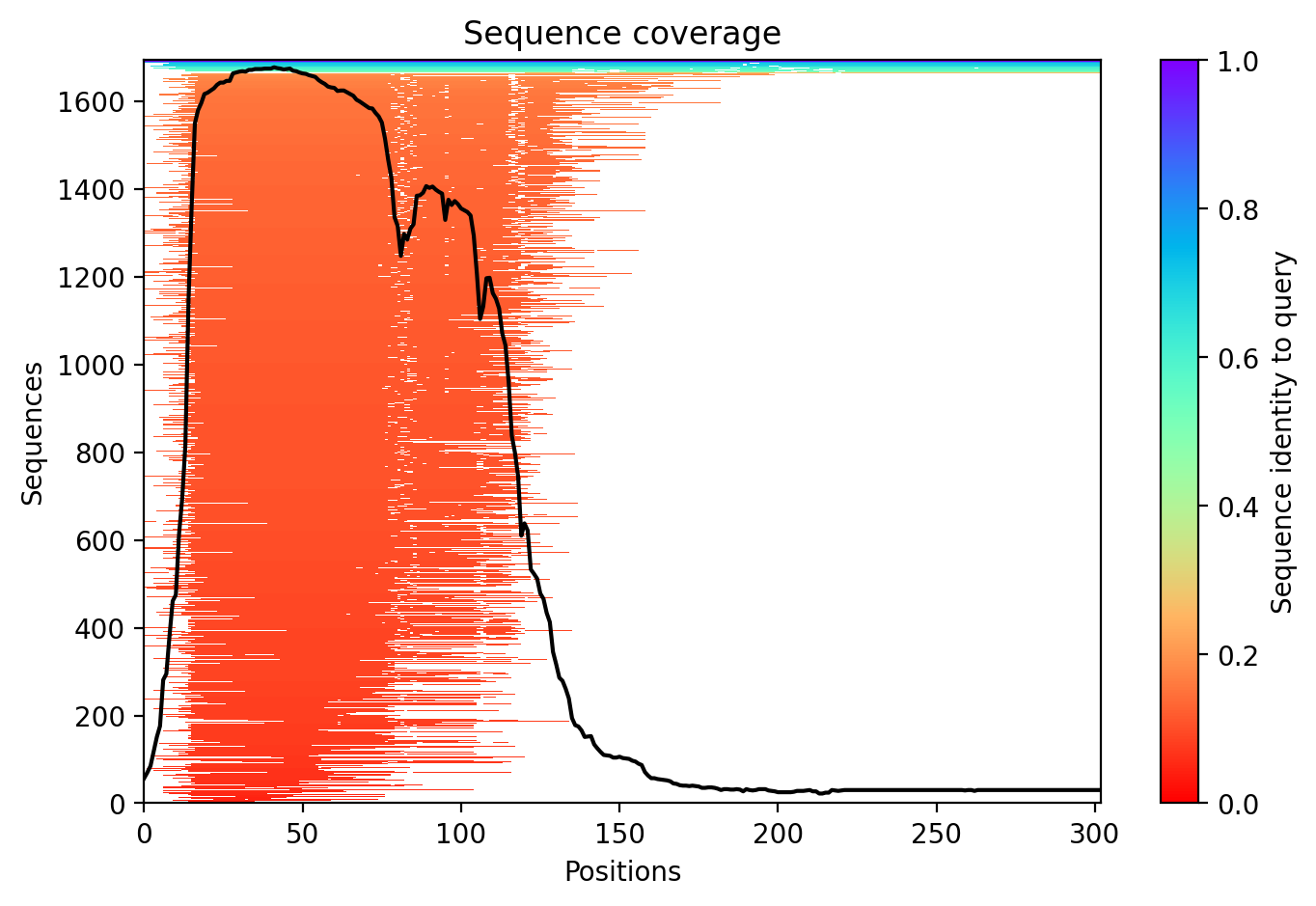

Supplement: Supplementary file 5 — Supplementary Dataset 1 [file 41467_2024_53986_MOESM5_ESM.zip › Archaellum_Homologues_2024/MsedA_1348_Metallosphaera/MsedA_1348_Metallosphaera_ad736_coverage.png]

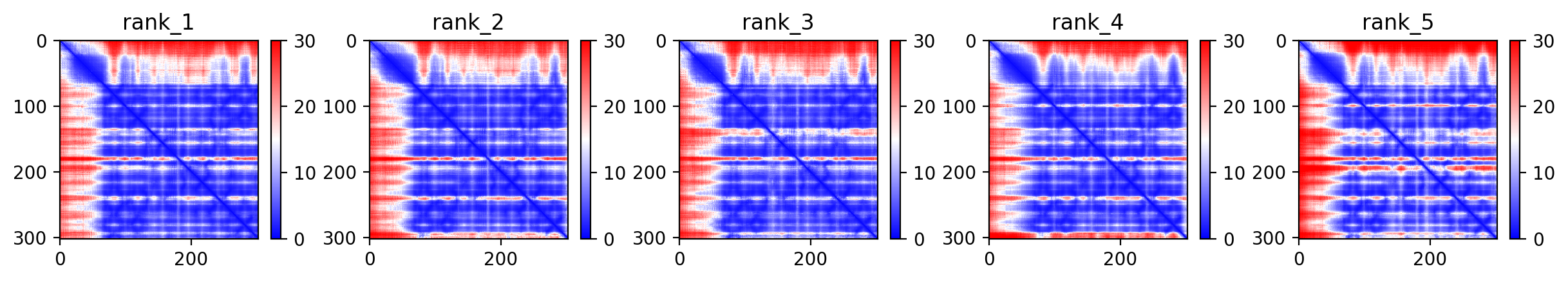

Supplement: Supplementary file 5 — Supplementary Dataset 1 [file 41467_2024_53986_MOESM5_ESM.zip › Archaellum_Homologues_2024/MsedA_1348_Metallosphaera/MsedA_1348_Metallosphaera_ad736_pae.png]

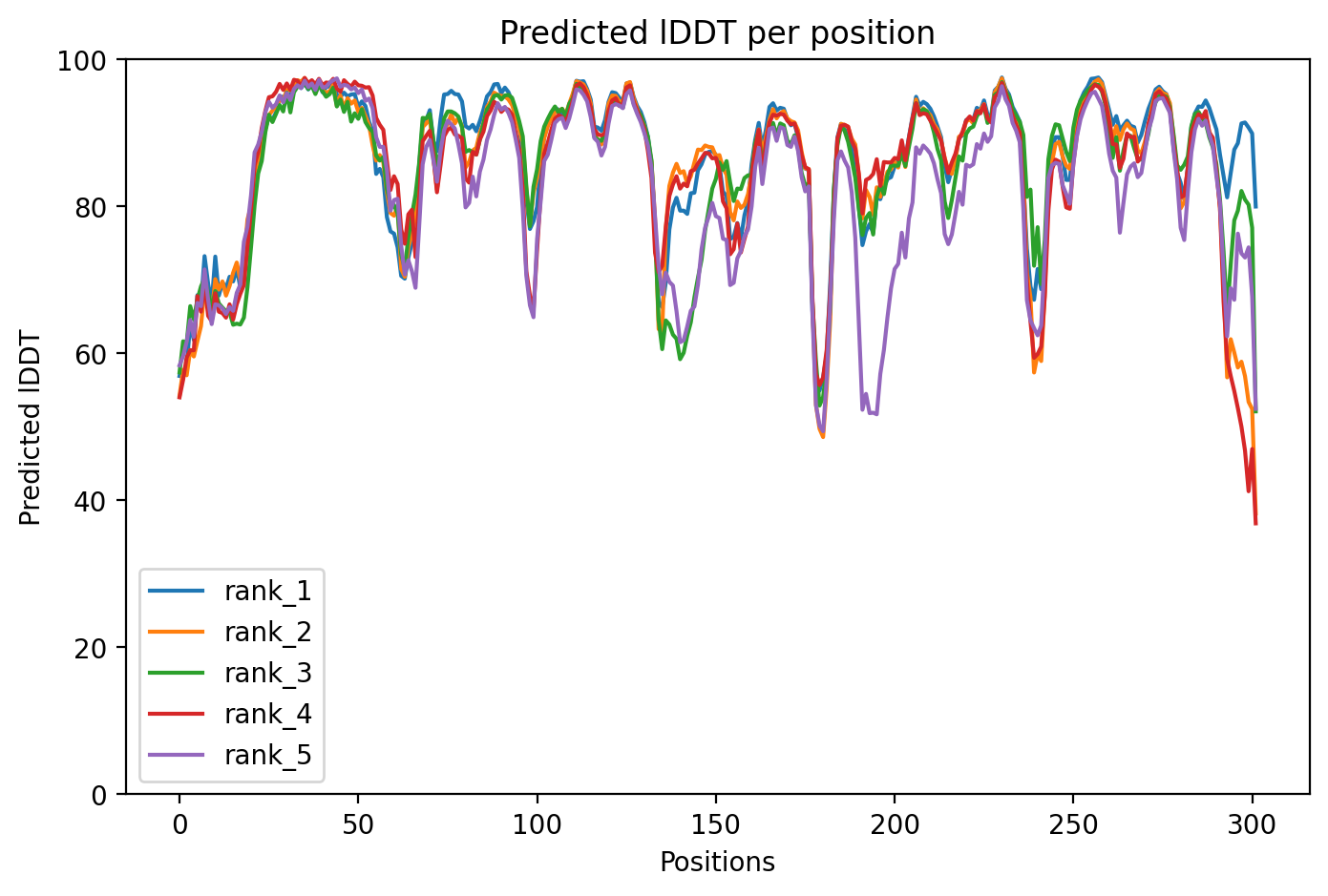

Supplement: Supplementary file 5 — Supplementary Dataset 1 [file 41467_2024_53986_MOESM5_ESM.zip › Archaellum_Homologues_2024/MsedA_1348_Metallosphaera/MsedA_1348_Metallosphaera_ad736_plddt.png]

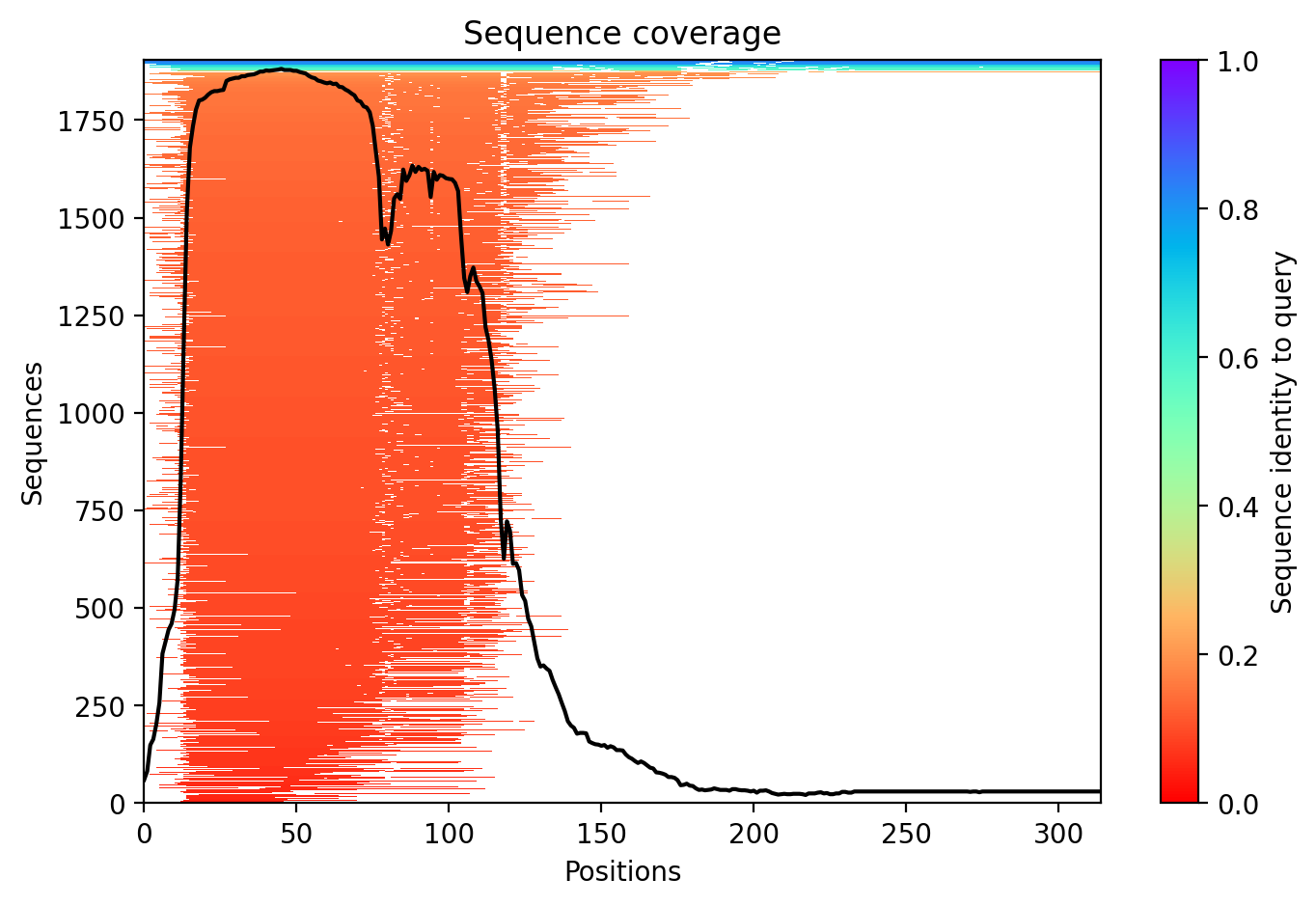

Supplement: Supplementary file 5 — Supplementary Dataset 1 [file 41467_2024_53986_MOESM5_ESM.zip › Archaellum_Homologues_2024/SACC_19750_Saccharolobus_caldissimus/SACC_19750_Saccharolobus_caldissimus_d4f34_coverage.png]

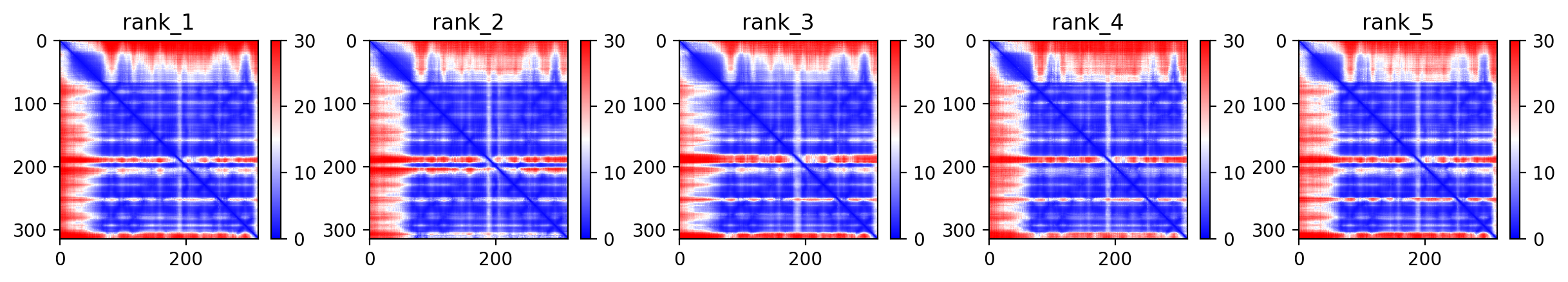

Supplement: Supplementary file 5 — Supplementary Dataset 1 [file 41467_2024_53986_MOESM5_ESM.zip › Archaellum_Homologues_2024/SACC_19750_Saccharolobus_caldissimus/SACC_19750_Saccharolobus_caldissimus_d4f34_pae.png]

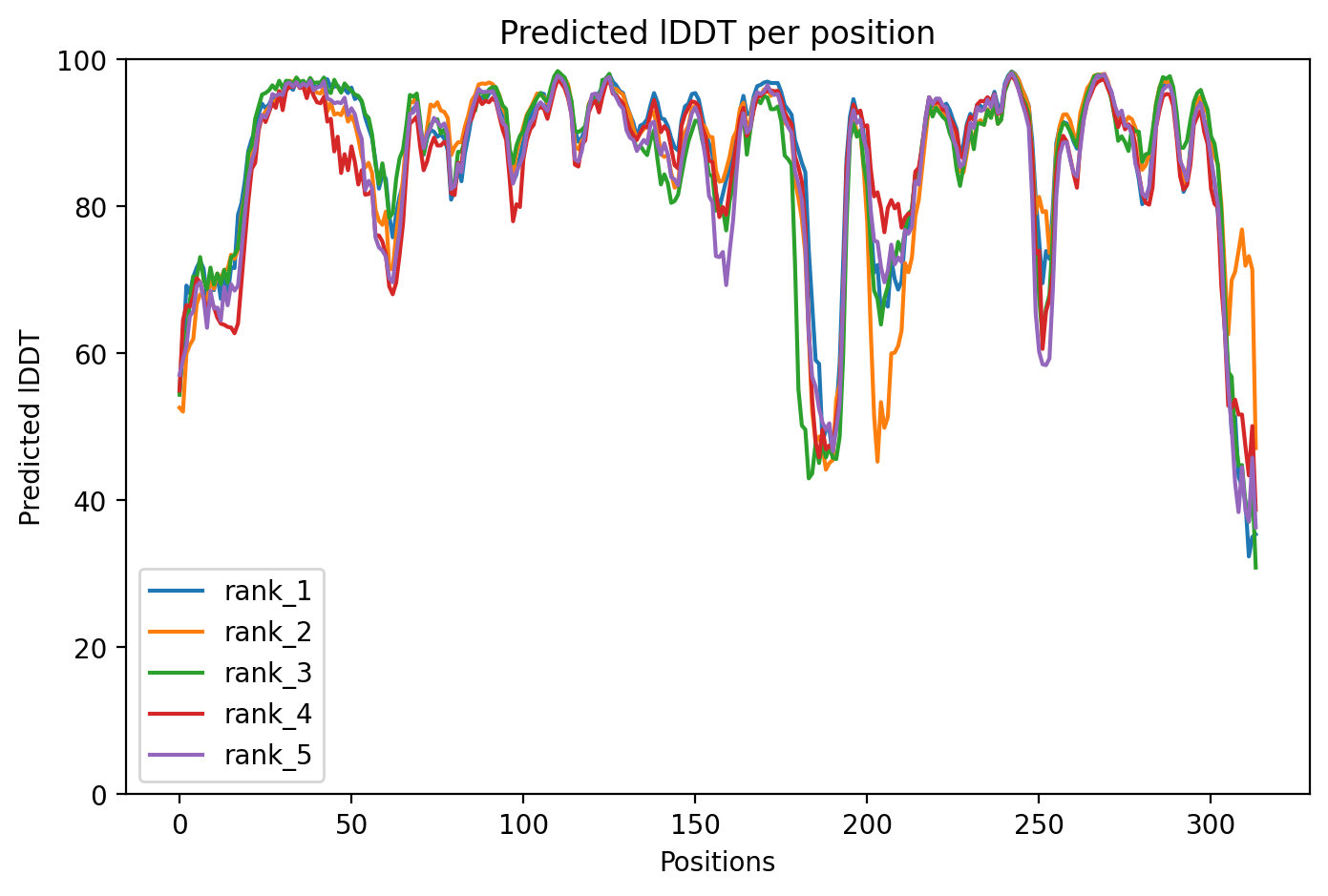

Supplement: Supplementary file 5 — Supplementary Dataset 1 [file 41467_2024_53986_MOESM5_ESM.zip › Archaellum_Homologues_2024/SACC_19750_Saccharolobus_caldissimus/SACC_19750_Saccharolobus_caldissimus_d4f34_plddt.png]

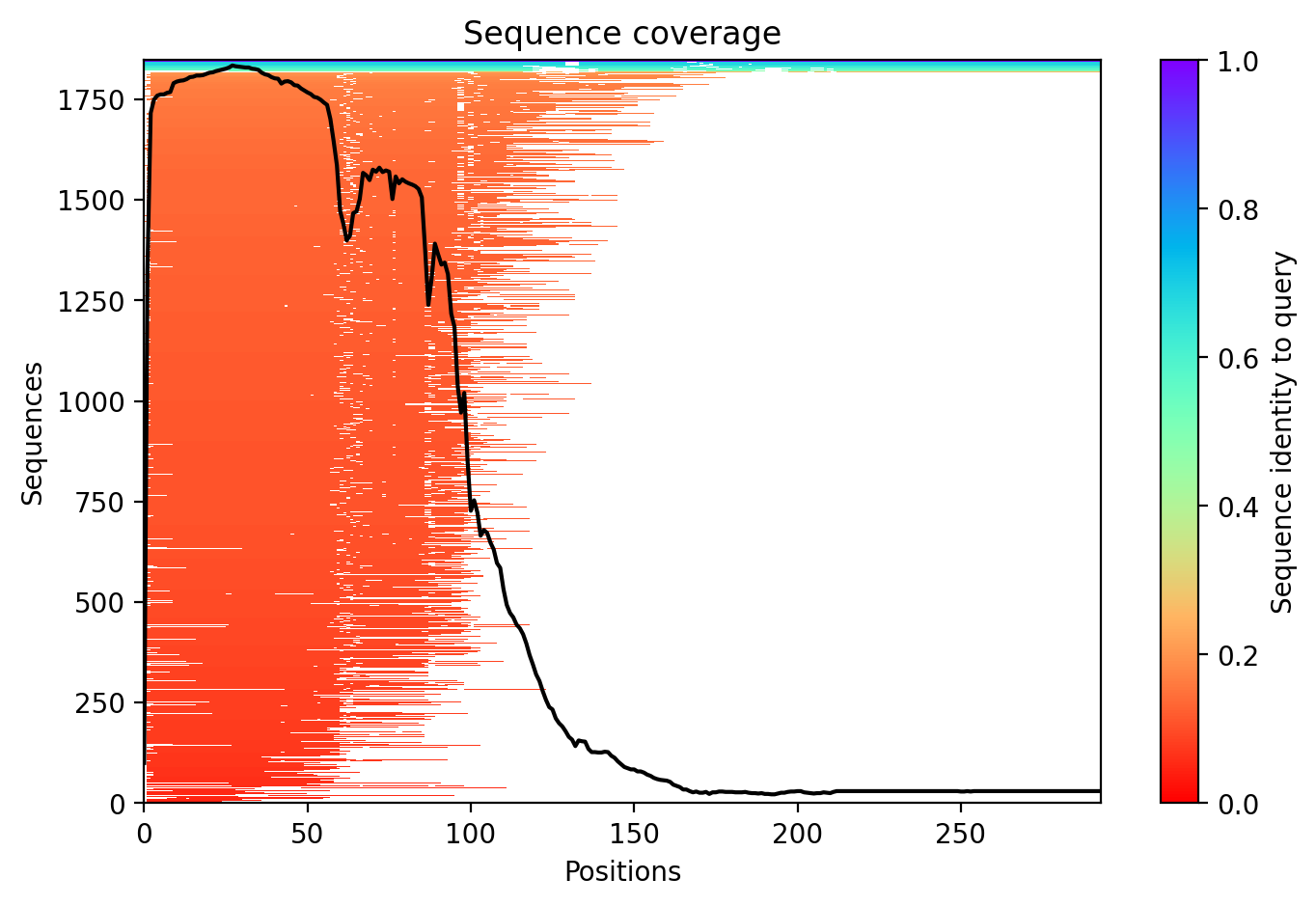

Supplement: Supplementary file 5 — Supplementary Dataset 1 [file 41467_2024_53986_MOESM5_ESM.zip › Archaellum_Homologues_2024/Saci_1178_Sulfolobus_Acidocaldarius/Saci_1178_Sulfolobus_Acidocaldarius_b2d98_coverage.png]

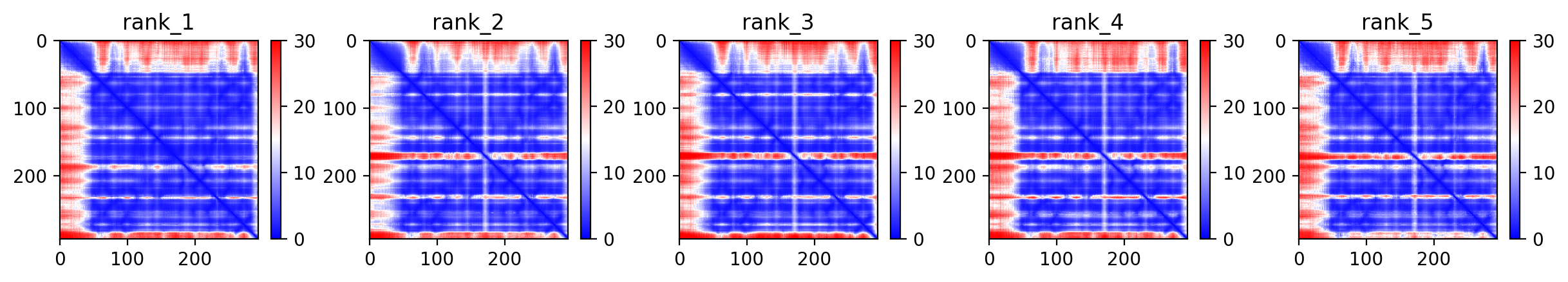

Supplement: Supplementary file 5 — Supplementary Dataset 1 [file 41467_2024_53986_MOESM5_ESM.zip › Archaellum_Homologues_2024/Saci_1178_Sulfolobus_Acidocaldarius/Saci_1178_Sulfolobus_Acidocaldarius_b2d98_pae.png]

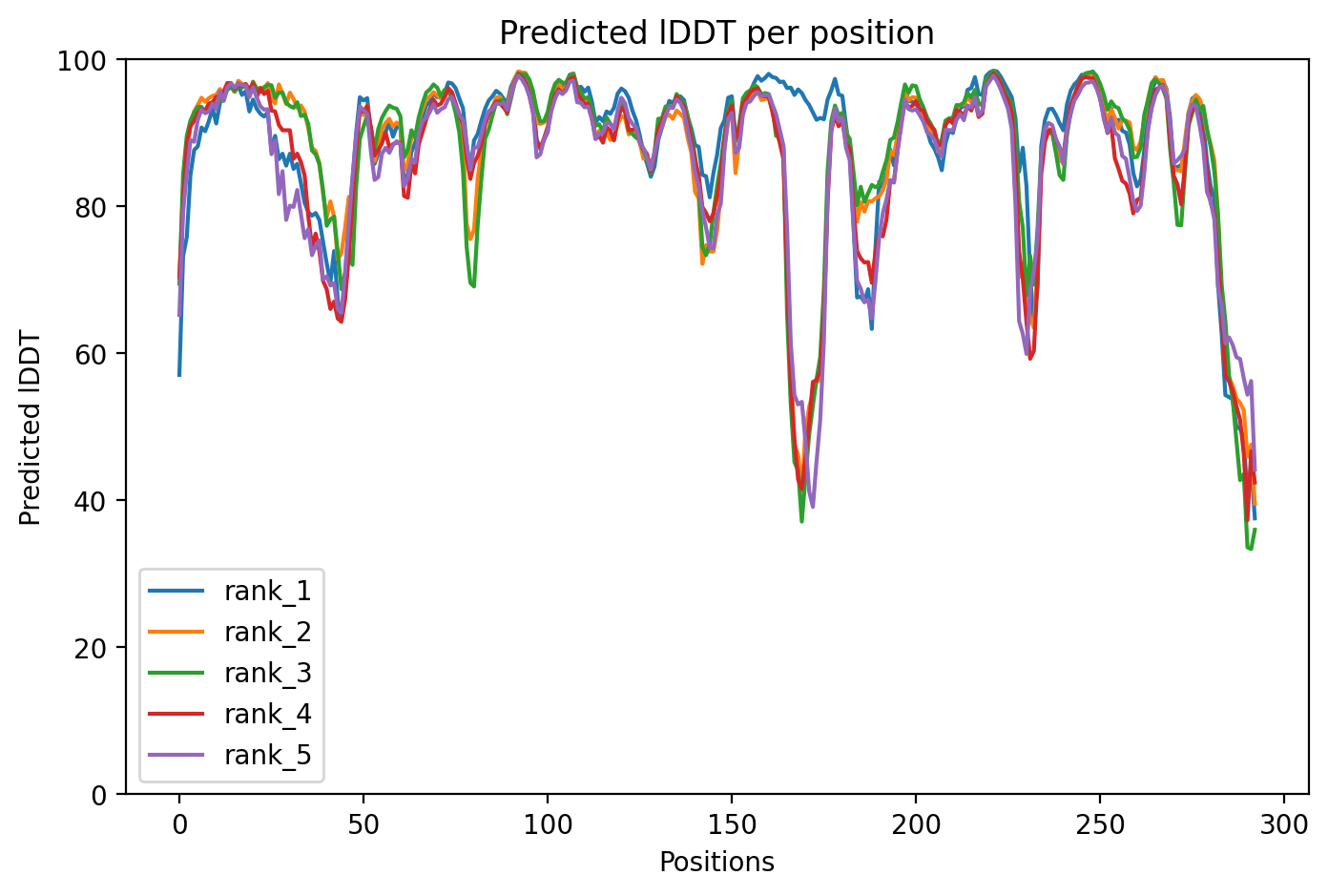

Supplement: Supplementary file 5 — Supplementary Dataset 1 [file 41467_2024_53986_MOESM5_ESM.zip › Archaellum_Homologues_2024/Saci_1178_Sulfolobus_Acidocaldarius/Saci_1178_Sulfolobus_Acidocaldarius_b2d98_plddt.png]

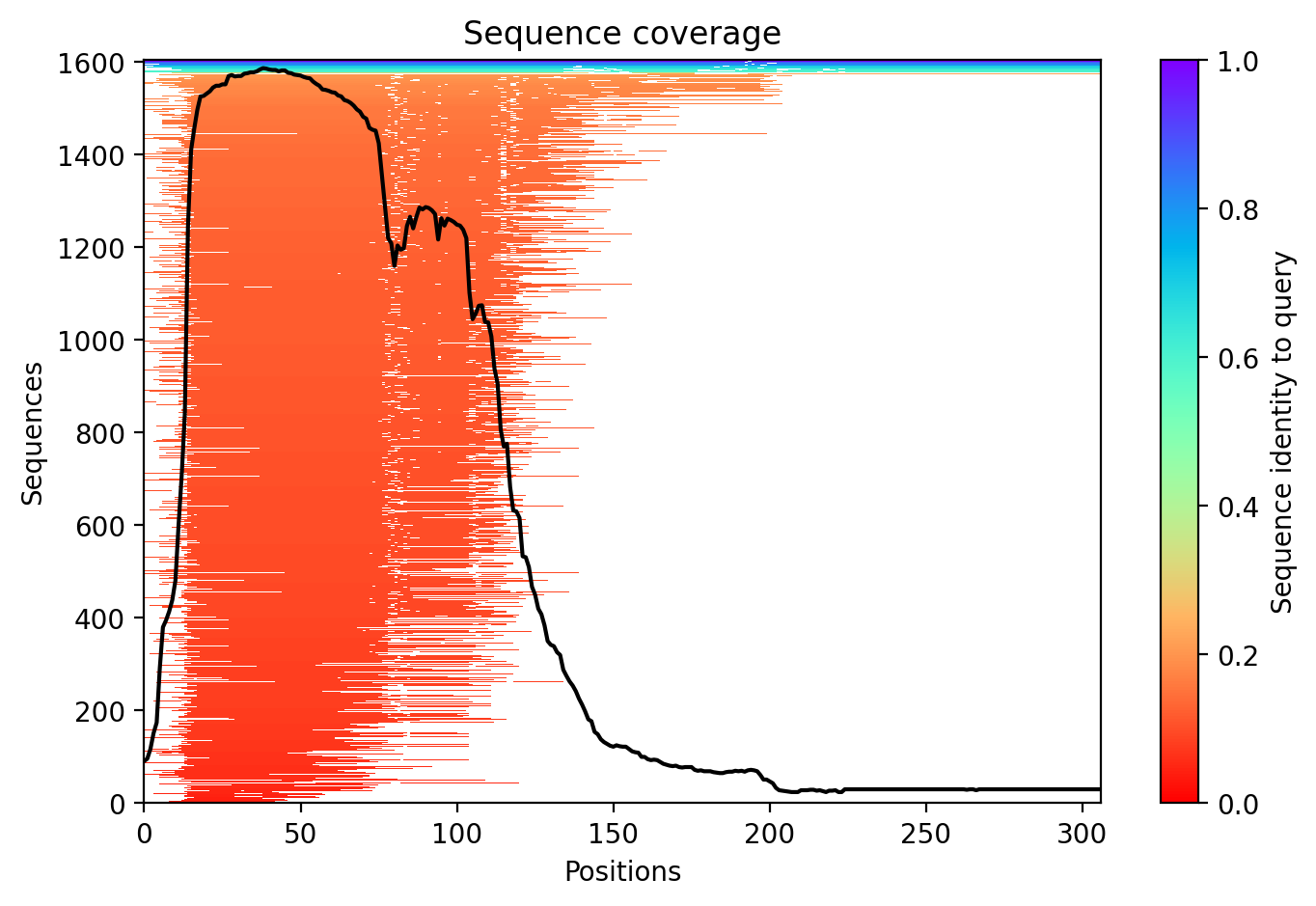

Supplement: Supplementary file 5 — Supplementary Dataset 1 [file 41467_2024_53986_MOESM5_ESM.zip › Archaellum_Homologues_2024/SiRe_0124_Sulfolobus_islandicus/SiRe_0124_Sulfolobus_islandicus_95109_coverage.png]

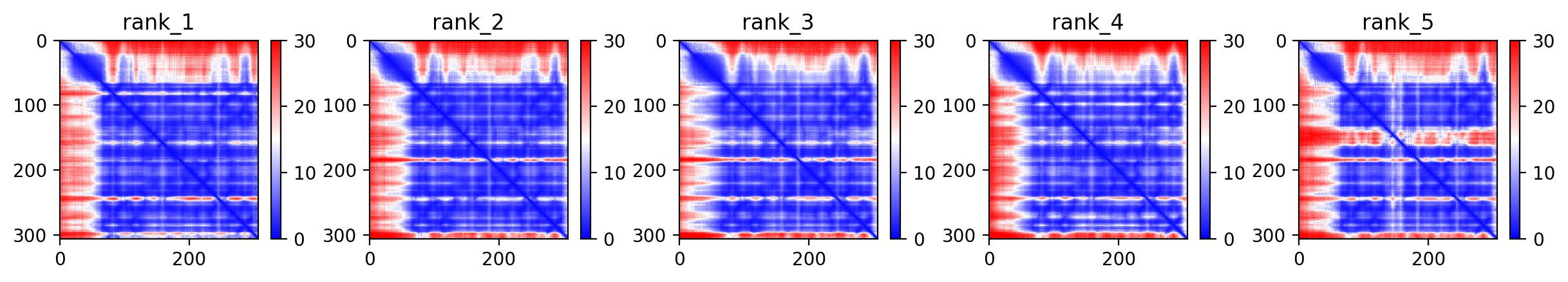

Supplement: Supplementary file 5 — Supplementary Dataset 1 [file 41467_2024_53986_MOESM5_ESM.zip › Archaellum_Homologues_2024/SiRe_0124_Sulfolobus_islandicus/SiRe_0124_Sulfolobus_islandicus_95109_pae.png]

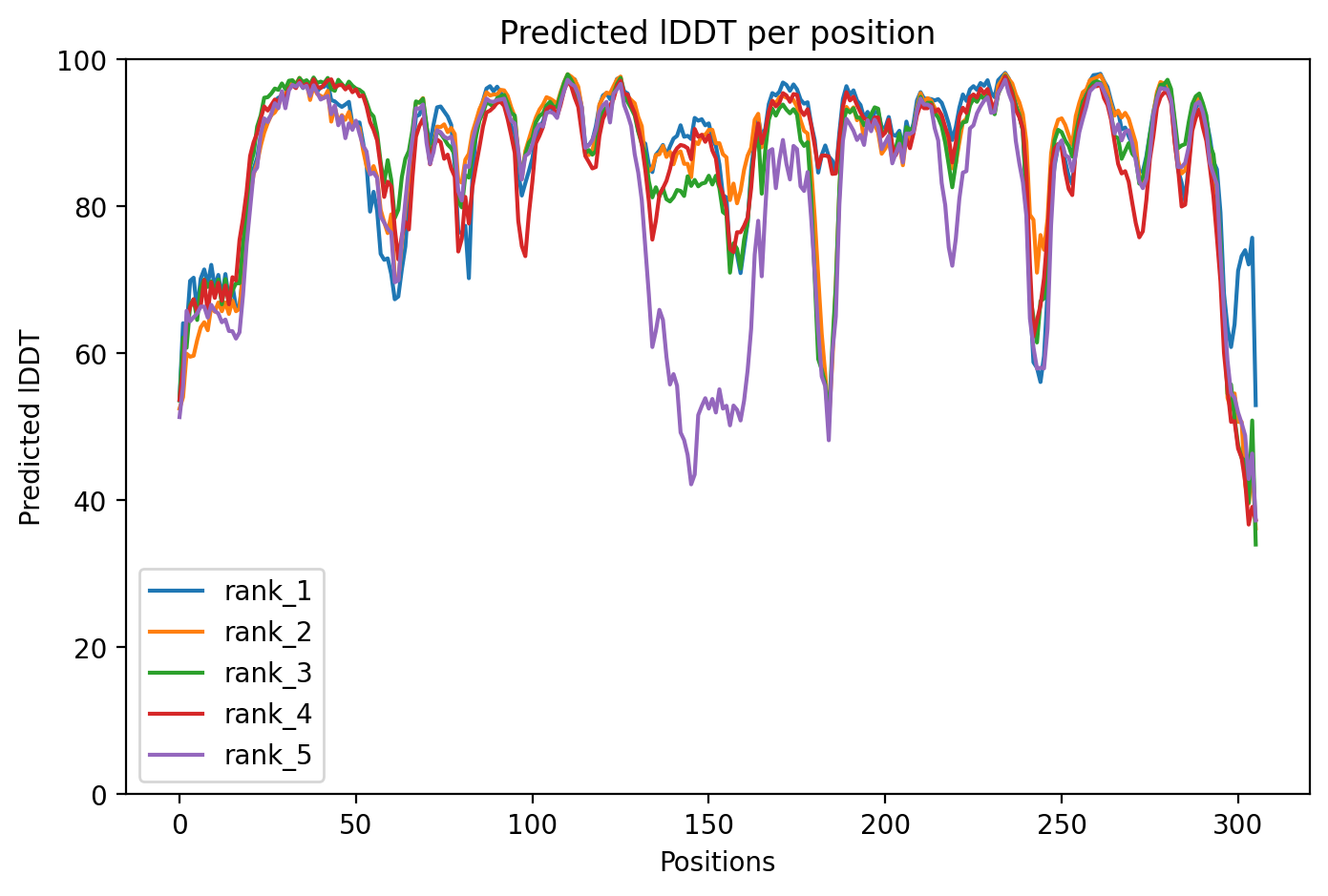

Supplement: Supplementary file 5 — Supplementary Dataset 1 [file 41467_2024_53986_MOESM5_ESM.zip › Archaellum_Homologues_2024/SiRe_0124_Sulfolobus_islandicus/SiRe_0124_Sulfolobus_islandicus_95109_plddt.png]

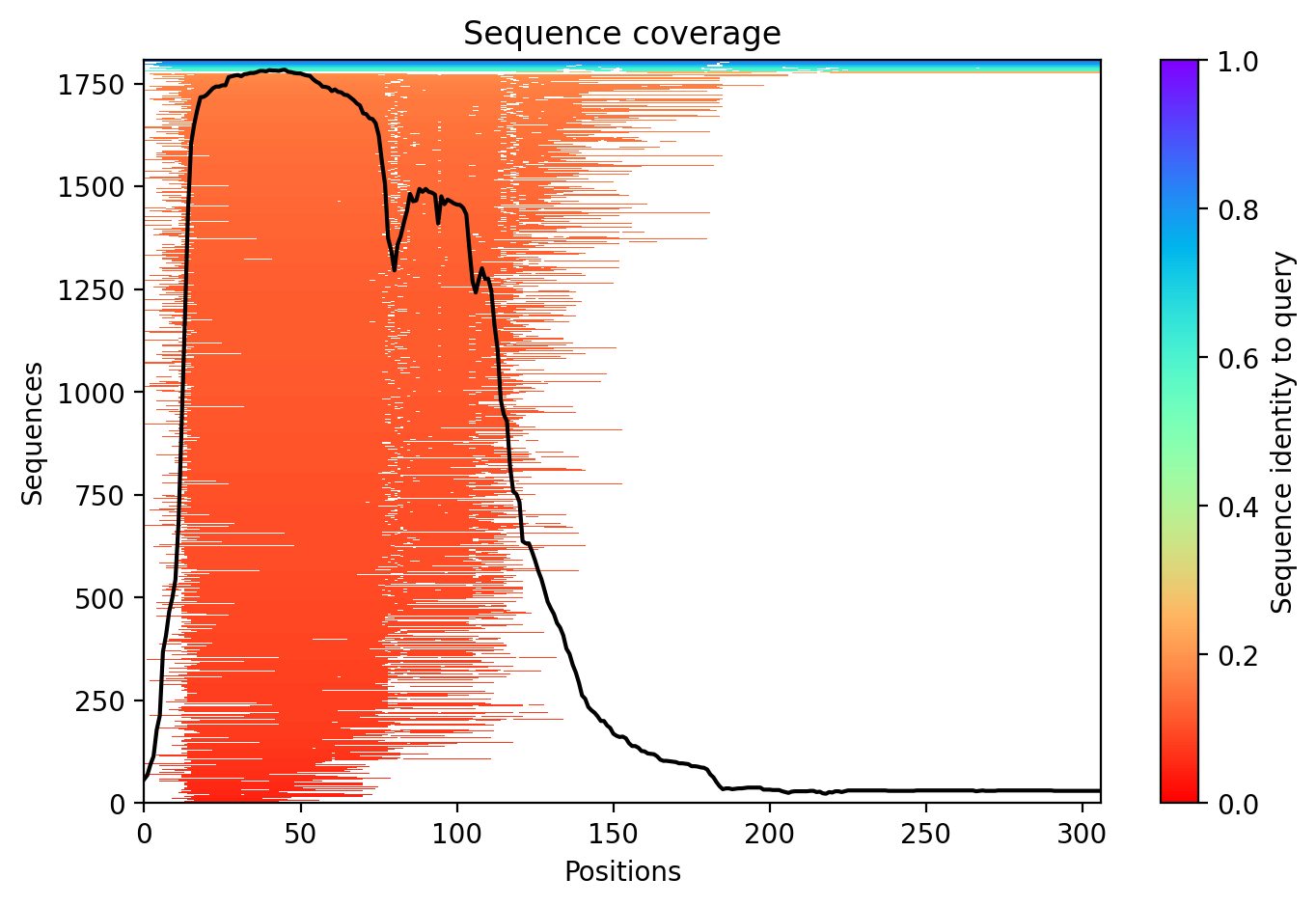

Supplement: Supplementary file 5 — Supplementary Dataset 1 [file 41467_2024_53986_MOESM5_ESM.zip › Archaellum_Homologues_2024/STK_25180_Sulfurisphaera_tokodaii/STK_25180_Sulfurisphaera_tokodaii_db704_coverage.png]

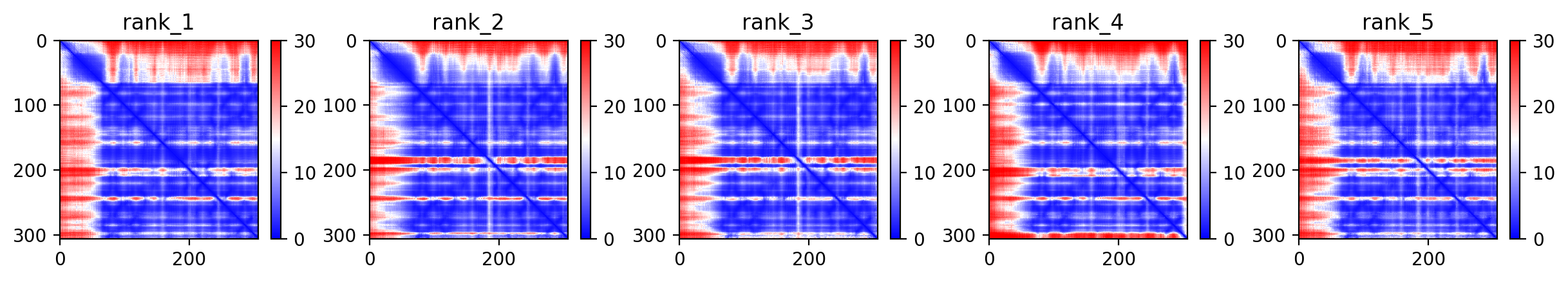

Supplement: Supplementary file 5 — Supplementary Dataset 1 [file 41467_2024_53986_MOESM5_ESM.zip › Archaellum_Homologues_2024/STK_25180_Sulfurisphaera_tokodaii/STK_25180_Sulfurisphaera_tokodaii_db704_pae.png]

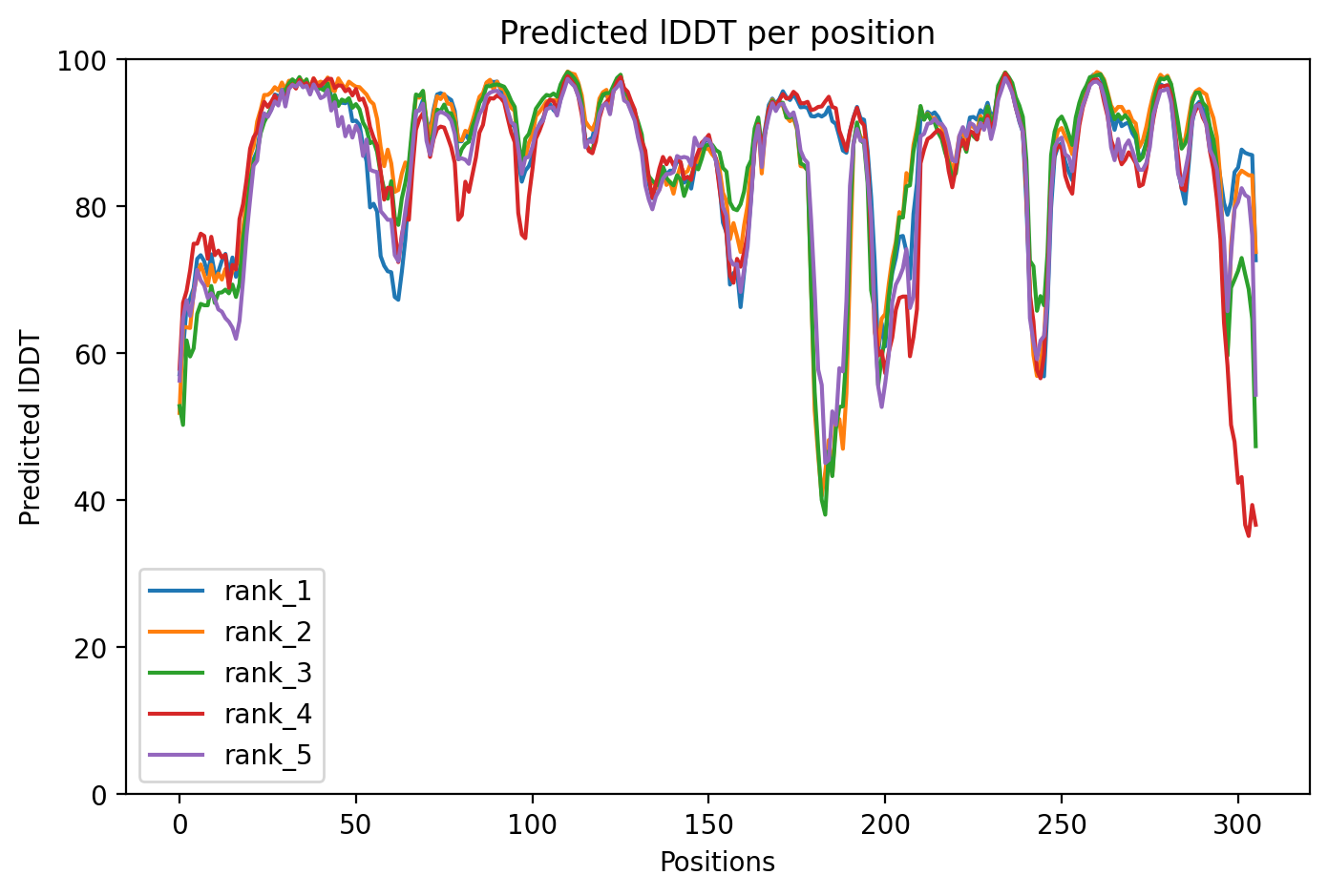

Supplement: Supplementary file 5 — Supplementary Dataset 1 [file 41467_2024_53986_MOESM5_ESM.zip › Archaellum_Homologues_2024/STK_25180_Sulfurisphaera_tokodaii/STK_25180_Sulfurisphaera_tokodaii_db704_plddt.png]
