# Supplementary material for: Towards a molecular picture of the archaeal cell surface
Source: Nat Commun. 2024 Nov 29;15:10401. doi: 10.1038/s41467-024-53986-9 (PMC11607397; doi:10.1038/s41467-024-53986-9)
Supplement: Supplementary file 6 — Reporting Summary [file 41467_2024_53986_MOESM6_ESM.pdf]

nature portfolio

Corresponding author(s): Bertram Daum

Last updated by author(s): 27/09/2024

Reporting Summary

Nature Portfolio wishes to improve the reproducibility of the work that we publish. This form provides structure for consistency and transparency in reporting. For further information on Nature Portfolio policies, see our Editorial Policies and the Editorial Policy Checklist.

Statistics

For all statistical analyses, confirm that the following items are present in the figure legend, table legend, main text, or Methods section.

n/a	Confirmed	
☐	☒	The exact sample size (*n*) for each experimental group/condition, given as a discrete number and unit of measurement
☒	☐	A statement on whether measurements were taken from distinct samples or whether the same sample was measured repeatedly
☒	☐	The statistical test(s) used AND whether they are one- or two-sided<br>*Only common tests should be described solely by name; describe more complex techniques in the Methods section.*
☒	☐	A description of all covariates tested
☒	☐	A description of any assumptions or corrections, such as tests of normality and adjustment for multiple comparisons
☐	☒	A full description of the statistical parameters including central tendency (e.g. means) or other basic estimates (e.g. regression coefficient) AND variation (e.g. standard deviation) or associated estimates of uncertainty (e.g. confidence intervals)
☒	☐	For null hypothesis testing, the test statistic (e.g. *F*, *t*, *r*) with confidence intervals, effect sizes, degrees of freedom and *P* value noted<br>*Give P values as exact values whenever suitable.*
☒	☐	For Bayesian analysis, information on the choice of priors and Markov chain Monte Carlo settings
☒	☐	For hierarchical and complex designs, identification of the appropriate level for tests and full reporting of outcomes
☒	☐	Estimates of effect sizes (e.g. Cohen's *d*, Pearson's *r*), indicating how they were calculated

Our web collection on statistics for biologists contains articles on many of the points above.

Software and code

Policy information about availability of computer code

Data collection	Data were collected using the EPU 1.10.0.65 software, running on a Titan Krios microscope.
Data analysis	For data analysis CryoSPARC 3.3.0 and 4.4.0, ChimeraX 1.4, ChimeraX 1.6, DeepEMhancer 0.11, SignalP5, PSI-Blast, REFMAC 5.8.0267, CCPEM 1.5.0, Alphafold2, ColabFold, Clustal Omega, MOLREP 11.7.03, Phenix 1.19.2-4158, Coot 0.9.6, JLigant 2.6 and CCP4 7.1 were used

For manuscripts utilizing custom algorithms or software that are central to the research but not yet described in published literature, software must be made available to editors and reviewers. We strongly encourage code deposition in a community repository (e.g. GitHub). See the Nature Portfolio guidelines for submitting code & software for further information.

Data

Policy information about availability of data

All manuscripts must include a data availability statement. This statement should provide the following information, where applicable:

- Accession codes, unique identifiers, or web links for publicly available datasets
- A description of any restrictions on data availability
- For clinical datasets or third party data, please ensure that the statement adheres to our policy

The cryoEM maps generated in this study have been deposited in the EM DataResource and the Protein Databank under the following accession codes. EMD-18700 (www.emdataresource.org/EMD-18700) / PDB 8QX4 (https://www.rcsb.org/structure/8QX4) for the wild type archaellum,
EMD-19608 (https://www.emdataresource.org/EMD-19608) / PDB 8RZL (https://www.rcsb.org/structure/8RZL) for the wild type thread,

EMD-19960 (https://www.emdataresource.org/EMD-19960) / PDB 9ETS (https://www.rcsb.org/structure/9ETS) for the wild type Aap,
EMD-19961 (https://www.emdataresource.org/EMD-19961) / PDB 9ETT (https://www.rcsb.org/structure/9ETT) for the Δagl3 archaellum,
and EMD-19990 (https://www.emdataresource.org/EMD-19960) / PDB 9EV0 (https://www.rcsb.org/structure/9EV0) for the Δagl3 Aap.
Previously published structures used for comparison can be found in the PDB databank (https://www.rcsb.org) under the following accession codes: PDB-8CWM (https://doi.org/10.2210/pdb8CWM/pdb) for the S. islandicus archaellum 60; PDB-7OFQ (https://doi.org/10.2210/pdb7OFQ/pdb) for the M. villosus archaellum 34; PDB-5Z1L (https://doi.org/10.2210/pdb5Z1L/pdb) for the M. maripaludis archaellum 33; PDB-5O4U (https://doi.org/10.2210/pdb5O4U/pdb) for the P. furiosus archaellum 32; PDB-5TFY (https://doi.org/10.2210/pdb5TFY/pdb) for the M. hungatei archaellum. 31
The raw image data used in this study have been deposited to the Electron Microscopy Public Image Archive (EMPIAR) under accession numbers EMPIAR-12184 (https://www.ebi.ac.uk/empiar/EMPIAR-12184/) for the wild type filaments isolated from strain MW2106 and EMPIAR-12196 (https://www.ebi.ac.uk/empiar/EMPIAR-12196/) for the Δagl3 mutant filaments from strain MW039. The S. acidocaldarius (DSM639) genome can be accessed via the KEGG accession code T00251 (https://www.genome.jp/entry/gn:T00251) or the NCBI gene bank code CP000077 112 (https://www.ncbi.nlm.nih.gov/nuccore/CP000077,112). The transcriptomics data analysed in this study can be accessed in the Pan Genomic Database for Genomic Elements Toxic To Bacteria under the following link: https://exploration.weizmann.ac.il/TCOL/index_singleOrg.php?organism=sulfolobus_acidocaldarius&tab=0.

Research involving human participants, their data, or biological material

Policy information about studies with human participants or human data. See also policy information about sex, gender (identity/presentation), and sexual orientation and race, ethnicity and racism.

Reporting on sex and gender	n/a
Reporting on race, ethnicity, or other socially relevant groupings	n/a
Population characteristics	n/a
Recruitment	n/a
Ethics oversight	n/a

Note that full information on the approval of the study protocol must also be provided in the manuscript.

Field-specific reporting

Please select the one below that is the best fit for your research. If you are not sure, read the appropriate sections before making your selection.

[x] Life sciences [ ] Behavioural & social sciences [ ] Ecological, evolutionary & environmental sciences

For a reference copy of the document with all sections, see nature.com/documents/nr-reporting-summary-flat.pdf

Life sciences study design

All studies must disclose on these points even when the disclosure is negative.

Sample size	For single particle processing, the number of particles used in the final structure was determined from the original particles extracted from micrographs. Then 2D classification followed by 3D refinements. This resulted in the following numbers of helical segments in the final reconstructions: Archaellum wt: 1,059,736; thread wt: 626,078; Aap wt: 505,862; archaellum Δagl3: 256,869; Aap Δagl3: 691,479.<br>Negative stain images presented are representative examples of dozens of micrographs of at least 3 replicates for each sample. As these images have merely been used for qualitative descriptions of cell morphology, no statistical analysis has been applied.
Data exclusions	No data were excluded
Replication	CryoEM structures are averages of hundreds of thousands of protein particles. The 3D maps undergo vigorous validation complying to standards widely accepted in the field.<br>The image pipeline is iterative, meaning 3D maps are reproduced several times. The iterative nature of cryoEM image processing is a robust means of verifying reproducibility. There were no instances where the CryoEM map could not be reproduced in this process, as the data continuously improved in resolution.
Randomization	Randomization was not relevant to this study, as statistical analyses were not performed
Blinding	Blinding was not relevant to this study, as statistical analyses were not performed

Reporting for specific materials, systems and methods

We require information from authors about some types of materials, experimental systems and methods used in many studies. Here, indicate whether each material, system or method listed is relevant to your study. If you are not sure if a list item applies to your research, read the appropriate section before selecting a response.

Materials & experimental systems

n/a	Involved in the study
☒	☐ Antibodies
☒	☐ Eukaryotic cell lines
☒	☐ Palaeontology and archaeology
☒	☐ Animals and other organisms
☒	☐ Clinical data
☒	☐ Dual use research of concern
☒	☐ Plants

Methods

n/a	Involved in the study
☒	☐ ChIP-seq
☒	☐ Flow cytometry
☒	☐ MRI-based neuroimaging

Plants

Seed stocks	*Report on the source of all seed stocks or other plant material used. If applicable, state the seed stock centre and catalogue number. If plant specimens were collected from the field, describe the collection location, date and sampling procedures.*
Novel plant genotypes	*Describe the methods by which all novel plant genotypes were produced. This includes those generated by transgenic approaches, gene editing, chemical/radiation-based mutagenesis and hybridization. For transgenic lines, describe the transformation method, the number of independent lines analyzed and the generation upon which experiments were performed. For gene-edited lines, describe the editor used, the endogenous sequence targeted for editing, the targeting guide RNA sequence (if applicable) and how the editor was applied.*
Authentication	*Describe any authentication procedures for each seed stock used or novel genotype generated. Describe any experiments used to assess the effect of a mutation and, where applicable, how potential secondary effects (e.g. second site T-DNA insertions, mosiacism, off-target gene editing) were examined.*
